# N,N-dimethylacetamide targets neuroinflammation in Alzheimer’s disease in in-vitro and ex-vivo models

**DOI:** 10.1038/s41598-023-34355-w

**Published:** 2023-05-01

**Authors:** Zeng-Hui Wei, Jagadish Koya, Nikita Acharekar, Jesus Trejos, Xing-Duo Dong, Francis A. Schanne, Charles R. Ashby, Sandra E. Reznik

**Affiliations:** 1 Department of Pharmaceutical Sciences, St. John’s University, Queens, NY 11439 USA; 2 Departments of Pathology and Obstetrics and Gynecology and Women’s Health, The University Hospital for Albert Einstein College of Medicine, Montefiore Medical Center, Bronx, NY 10461 USA

**Keywords:** Drug safety, Pharmacology, Drug discovery, Drug delivery

## Abstract

Alzheimer’s disease (AD) is a chronic degenerative brain disorder with no clear pathogenesis or effective cure, accounting for 60–80% of cases of dementia. In recent years, the importance of neuroinflammation in the pathogenesis of AD and other neurodegenerative disorders has come into focus. Previously, we made the serendipitous discovery that the widely used drug excipient N,N-dimethylacetamide (DMA) attenuates endotoxin-induced inflammatory responses in vivo. In the current work, we investigate the effect of DMA on neuroinflammation and its mechanism of action in in-vitro and ex-vivo models of AD. We show that DMA significantly suppresses the production of inflammatory mediators, such as reactive oxygen species (ROS), nitric oxide (NO) and various cytokines and chemokines, as well as amyloid-β (Aβ), in cultured microglia and organotypic hippocampal slices induced by lipopolysaccharide (LPS). We also demonstrate that DMA inhibits Aβ-induced inflammation. Finally, we show that the mechanism of DMA’s effect on neuroinflammation is inhibition of the nuclear factor kappa-B (NF-κB) signaling pathway and we show how DMA dismantles the positive feedback loop between NF-κB and Aβ synthesis. Taken together, our findings suggest that DMA, a generally regarded as safe compound that crosses the blood brain barrier, should be further investigated as a potential therapy for Alzheimer’s disease and neuroinflammatory disorders.

## Introduction

Neuroinflammation is an inflammatory response within the brain or spinal cord, which is mediated by expression of inflammatory mediators produced primarily by microglia in the central nervous system (CNS)^1^. Chronic and uncontrolled neuroinflammation causes excessive microglial activation, resulting in release of inflammatory mediators in the brain and eventually damage to the nervous system^2^. Neuroinflammation is also characteristic of neurodegenerative diseases like Alzheimer’s disease (AD)^3–5^. AD, named after German pathologist Alois Alzheimer^6,7^, mostly appears in people who are over 65 years old with progressive cognitive and behavioral impairment^8–11^. Its increasing global incidence and resistance to treatment make AD the sixth leading cause of death in the United States (US)^12–14^. To date, the cause of AD is not well understood^15^, but there is an increasing appreciation that neuroinflammation and the pathogenesis of AD are closely intertwined^16^. In the past few decades, many treatments targeting the two cellular hallmarks of AD, amyloid-β (Aβ) and tau proteins, have failed due to toxicity and/or lack of efficacy^17–22^. Meanwhile, growing evidence suggests that chronic microglial activation induced by neuroinflammation plays a key role in the progression of AD, especially in accumulation of Aβ in the brain^23–26^. Therefore, anti-inflammatory therapeutic approaches to modify AD progression are the basis for ongoing and future therapeutic trials in this area, a trend reflected by the AD drug development pipeline in the 2021 US Food and Drug Administration (FDA) registry in which 12% of agents in Phase III trials, 19% of agents in Phase II trials and 22% of agents in Phase I trials targeted inflammation to modify AD progression^27^.

Activation of toll-like receptors (TLRs) induced by neuronal stimulation (e.g., lipopolysaccharide (LPS) or Aβ) triggers a series of intracellular inflammatory reactions controlled by the nuclear factor kappa-B (NF-κB) signaling pathway^28,29^. These intracellular events lead to activation of the inhibitor kappa B kinase (IKK), resulting in phosphorylation of inhibitor kappa B (IκB) and its subsequent degradation by the proteasome. Degradation of IκB leads to release of NF-κB dimers (prototypically p65/p50 subunits), their translocation into the nucleus and their binding to consensus sequences of neuronal gene targets like amyloid-beta precursor protein (APP)^29,30^. APP is best known as the precursor molecule of Aβ, which forms when APP is consecutively cleaved by the enzymes β-secretase and γ-secretase^8,11^. Moreover, activation of NF-κB pathways stimulates a cascade of production of inflammatory mediators including reactive oxygen species (ROS), nitric oxide (NO) and pro-inflammatory cytokines and chemokines. Increased levels of pro-inflammatory cytokines, such as tumor necrosis factor (TNF)-α, interleukin (IL)-6 and IL-1β, are believed to inhibit phagocytosis of Aβ and promote phosphorylation and cleavage of APP, thereby exacerbating accumulation of Aβ in AD brains^31–35^.

Previous studies have shown that N,N-dimethylacetamide (DMA), a common organic solvent and FDA approved drug excipient, significantly inhibits inflammatory responses in in-vivo models of preterm birth^36^, inflammatory bowel disease^37^, osteoporosis^38^ and obesity^39^ via inhibition of the NF-κB signaling pathway^29^. DMA is a small molecule with a molecular weight of 87 g/mol, which can easily penetrate the blood–brain barrier^40,41^. The aim of the current study was to (1) investigate the anti-neuroinflammatory effects of DMA in vitro and ex vivo and (2) elucidate the mechanism of DMA’s anti-neuroinflammatory actions.

## Materials and methods

### Cell culture

The mouse microglial cell line SIM-A9 and human microglial cell line HMC3 (American Type Culture Collection, ATCC) were used in this study. SIM-A9 cells were cultured in Dulbecco’s Modified Eagle Medium: Nutrient Mixture F-12 (Ham’s) (DMEM: F-12) (ATCC) containing 10% heat-inactivated fetal bovine serum (FBS) (Gibco), 5% heat-inactivated horse serum (HS) (ATCC) and 1% penicillin/streptomycin (Gibco). HMC3 cells were cultured in Eagle’s Minimal Essential Medium (EMEM) (Quality Biological) and supplemented with 10% heat-inactivated FBS and 1% penicillin/streptomycin (Gibco). All cells were maintained in an incubator set at 37 °C and 5% CO_2_ and allowed to grow to 80–90% confluency before being sub-cultured or used in experiments.

### Preparation of Aβ42 oligomers

Aβ42 peptide (Invitrogen) was prepared as per the manufacturer’s protocol. Briefly, 1 mg of Aβ42 was dissolved in 1 ml of 1,1,1,3,3,3-Hexafluoro-2-propanol (HFIP) (Sigma-Aldrich) and incubated for 1 h. After incubation, the HFIP was removed and dimethyl sulfoxide (DMSO) (ATCC) was added to resolve Aβ42 at a 1 mg/ml concentration. The solution was diluted to 100 μM with Minimum Essential Medium (MEM) (Corning) and incubated at 4 °C for 24 h. After 24-h incubation, the solution was centrifuged at 4 °C and 16,000×*g* for 10 min. The supernatant containing Aβ42 oligomers was used for experiments following established protocols^42,43^.

### Cell viability assay

HMC3 and SIM-A9 cells were seeded at 20,000 and 25,000 cells/well, respectively, in 96-well plates. After overnight incubation, the cells were incubated in the absence or presence of incremental concentrations (0.1–100 mM) of DMA for 2 h. To mimic experimental conditions, in which cells are stimulated with either LPS or Aβ, HMC3 cells and SIM-A9 cells were incubated with 1 μg/mL LPS (*Escherichia coli* 026:B6) (Sigma-Aldrich) or 3 μM Aβ42 for an additional 24 h, respectively. The concentration of 1 μg/mL LPS was chosen based on our long experience with eliciting cytokine secretion from cultured cells with this serotype of LPS^29^. The concentration of 3 μM Aβ42 was chosen based on reports of Aβ42 in this concentration range eliciting cellular responses^44^. To address the possibility that DMA’s toxicity is affected by the absence or presence of each of these stimulants, we performed the cell viability assays in the absence and presence of LPS and Aβ. Cell viability was evaluated by performing the MTT (3-(4,5-dimethylthiazol-2-yl)-2,5-diphenyltetrazolium bromide) assay. The MTT reagent was added to a final concentration of 0.5% and incubated further for 2 h at 37 °C. Subsequently, cell culture media was aspirated and 100 µL of DMSO (BDH) was added to each well to dissolve the formed purple formazan crystals. The absorbance of the resulting purple solution was measured at a wavelength of 570 nm. Three independent experiments were performed in triplicate.

### Griess assay

Nitrite (NO^−2^) is one of the primary inert end products of nitric oxide (NO) metabolism in biological systems^42^. NO^−2^ levels in cell culture supernatants were measured by Griess assay (Promega) to track levels of NO formation indirectly. Samples were prepared by seeding 1.5 × 10^6^ SIM-A9 cells in 25 cm^2^ flasks and incubating the cells overnight. Cells were then incubated in the absence or presence of DMA (0.1–10 mM) and NF-κB inhibitor BAY 11-7082 (5 μM) (Enzo Lab Sciences) for 2 h. Subsequently, cells were incubated with 1 μg/mL LPS for 24 h. The supernatants were collected after centrifuging at 4 °C and 1000×*g* for 10 min and then subjected to Griess assay following the manufacturer’s instructions. Three independent experiments were performed in triplicate. A standard curve was created as per the manufacturer’s instructions to determine the concentration of NO^−2^ in the samples.

### Flow cytometry

Flow cytometry was performed to detect the production of ROS in microglia. To prepare the samples, SIM-A9 cells were seeded at 1.5 × 10^6^ cells per 25 cm^2^ flasks and incubated at 37 °C overnight. Cells were then incubated in the absence or presence of 10 mM DMA for 2 h followed by 24 h of incubation with 1 μg/mL LPS. Cells were collected and centrifuged at 10,000 rpm for 5 min. The supernatants were removed, and cells were stained with CellROX Oxidative Stress Reagents (Invitrogen) at 37 °C for 30 min. Samples were then centrifuged at 10,000 rpm for 2 min and washed with phosphate buffered saline (PBS) three times. Cellular fluorescent intensity was detected by BD Accuri C6 flow cytometer (BD Biosciences). Quantification of ROS expression was based on three independent experiments.

### In-vitro assays for secreted cytokines and chemokines

HMC3 cells and SIM-A9 cells were incubated in the absence or presence of DMA (0.1–10 mM) or BAY 11-7082 (5 μM) for 2 h followed by 24 h of 1 μg/mL LPS or 3 μM Aβ42 stimulation. Cell culture supernatants were collected and centrifuged at 4 °C and 1000×*g* for 10 min. Enzyme linked immunosorbent assay (ELISA) kits (Thermo Fisher) were used to determine the concentrations of Interleukin (IL)-6, IL-8, IL-1β, tumor necrosis factor (TNF)-α, chemokine (C–C motif) ligand 2 (CCL2), granulocyte–macrophage colony stimulating factor (GM-CSF) and IL-10 following the manufacturer’s instructions. Three independent experiments were conducted in duplicate. A second-order polynomial equation generated from a standard curve was used to determine the concentration of each cytokine/chemokine with GraphPad Prism 7 software, as instructed by the manufacturer.

### Quantification of Aβ42

Aβ42 levels in microglia cells were evaluated using ELISA (Invitrogen). To prepare the samples, cells were first incubated in the absence or presence of DMA (0.1–10 mM) for 2 h and then incubated with 1 μg/mL LPS for 24 h. Subsequently, cells were collected and lysed in radio immunoprecipitation assay (RIPA) lysis buffer (G-biosciences) with a protease and phosphatase inhibitor cocktail (Thermo Fisher) for 30 min on ice. After centrifugation at 4 °C and 14,000×*g* for 20 min, the supernatants were collected for further use. The Pierce Bicinchoninic Acid (BCA) Protein Assay Kit (Thermo Fisher) was used to quantify the protein in each sample. Equal amounts of protein were used to conduct the ELISA based on the manufacturer’s instructions. Three independent experiments were performed.

### Reverse transcription-polymerase chain reaction (RT-PCR)

Total RNA was extracted by using the RNeasy Mini kit (Qiagen) and quantified using a Pico200 Microliter UV/Vis Spectrophotometer (VWR). The pure RNA was reverse transcribed to cDNA with SuperScript II Reverse Transcription Kit (Invitrogen). The cDNA samples were further used in Reverse Transcription Polymerase Chain Reaction (RT-PCR) (Agilent Technologies). The 2^−ΔΔCq^ method was used to quantify gene expression and the glyceraldehyde 3-phosphate dehydrogenase (GAPDH) was used as the internal control^45^. The primer sequences used in this analysis are listed in Supplementary Table S1.

### Western blot assay

Cells were lysed in RIPA lysis buffer with a protease and phosphatase inhibitor cocktail over the course of 30 min on ice. Samples were then centrifuged at 4 °C and 14,000×*g* for 20 min. The supernatants were collected, and the protein concentrations were quantified using the BCA Protein Assay Kit. Equal amounts of total protein (30 µg) were resolved by 10% sodium dodecyl sulfate polyacrylamide gel electrophoresis (SDS-PAGE) and electrophoretically transferred onto polyvinylidene fluoride (PVDF) membranes. To conserve reagents, membranes were then cropped and portions of the membrane containing the proteins of interest were used for western blotting. The membranes were blocked with 5% non-fat milk at room temperature for 2 h followed by overnight incubation at 4 °C with primary antibodies (Supplementary Table S2) (Cell Signaling Technologies). The membranes were then incubated at room temperature for 2 h with a 1:1000 dilution of horse-radish peroxidase (HRP)-linked secondary antibody (Cell Signaling Technologies) in 5% non-fat milk. The blots were developed using the Pierce enhanced chemiluminescence (Thermo Fisher) and normalized using GAPDH. Quantification of immunoblot was conducted by ImageJ software and based on three independent experiments.

### Immunofluorescence (IF)

Cells were fixed with 4% paraformaldehyde for 15 min at room temperature after being washed with ice-cold PBS. Cells were then permeabilized with 0.25% Triton X-100 for 15 min and blocked with 6% bovine serum albumin (BSA) in PBS for 1 h at 37 °C. Fixed cells were incubated with monoclonal antibody against the NF-κB p65 protein (1:200) (Cell Signaling Technologies) overnight at 4 °C. Thereafter, the cells were further incubated with Alexa fluor 488 donkey anti-rabbit IgG (1:400) (Invitrogen) for 2 h at 37 °C. To visualize the nuclei, cells were counterstained with 4′,6-diamidino-2-phenylindole (DAPI) after being rinsed with PBS. The immunofluorescence images were generated using a Nikon TE-2000S fluorescence microscope (Nikon Instruments Inc).

### Lactate dehydrogenase (LDH) assay

Because the hippocampus comprises a heterogenous population of cells, it is not suited for MTT assay. Therefore, a Pierce lactate dehydrogenase (LDH) assay kit (Thermo Fisher) was used to evaluate the viability of hippocampal slices by measuring the amount of LDH released into the culture medium following an established protocol^46^. After incubating the slices in 50% MEM, 25% Hanks Balanced Salt Solution (with calcium and magnesium) and 25% horse serum in the absence or presence of 1 µg/ml LPS or 3 μM Aβ42 and 10 mM DMA, the culture medium was collected and centrifuged at 4 °C and 1000×*g* for 10 min just before the slices were homogenized. The supernatants were then used in an LDH assay according to the manufacturer’s instructions. Briefly, 50 μl of each supernatant were added in duplicate into a 96-well plate followed by mixing with 50 μl of Reaction Mixture. The reaction was run for 30 min at 37 °C and the stop solution was then added. The absorbance was measured at a wavelength of 490 nm. Three independent experiments were performed.

### Ex vivo assays for secreted cytokines and chemokines

All protocols related to ex vivo assays were carried out under approval of the St. John’s University Institutional Animal Care and Use Committee (Protocol Number 1912.1). The concentrations of cytokines and chemokines secreted from hippocampal organotypic slices, including IL-6, IL-8, IL-1β, TNF-α, CCL2, GM-CSF and IL-10, were detected by ELISA. Homogenates for ELISA were prepared by collecting both hippocampi from three adult male Sprague Dawley rats, 10–21 days old. Animals were sacrificed via decapitation and brains were immediately excised from the cranium. Whole hippocampi were carefully dissected out and placed in ice cold calcium and magnesium free Hanks balanced salt solution. Hippocampi were then placed on top of a 1.5% agar block sliced at a thickness of 200 microns using a Syskiyou Tissue Slicer (SKU 14240000E). Slices were manually separated and individually immersed in ice cold calcium and magnesium free Hanks balanced salt solution. Using a trimmed transfer pipette, slices were removed from the ice cold media and placed into 6-well plates containing 50% MEM, 25% Hanks Balanced Salt Solution (with calcium and magnesium) and 25% horse serum, pre-warmed to 37 °C. Excess calcium free and magnesium free media on the top of the membrane was removed. Before any treatments with LPS, Aβ42 or DMA, the tissue was equilibrated to 37 °C in a cell culture incubator for 1 h. After the 1-h equilibration, the slices were incubated in the media with or without 10 mM DMA for two hours. Hippocampal slices were then stimulated with either 1 µg/ml LPS or 3 μM Aβ42 for 15 h.

Slices were then homogenized in 0.6 mL ice-cold lysis buffer with a protease and phosphatase inhibitor cocktail using a Polytron homogenizer (VWR). The samples were homogenized for 2 min every 15 min over the course of 2 h following published procedures^36,47^. Thereafter, the homogenates were centrifuged at 4 °C and 10,000×*g* for 15 min, and the supernatants were collected. The BCA assay was used to quantify the protein concentration in the supernatant of each sample. Equal amounts of protein samples were used to perform the ELISA. Three independent experiments (from three different rats) were conducted in duplicate.

### Statistical analysis

All the data were analyzed using GraphPad Prism 7 software and the statistical significance of the differences among and between treatments was tested with one-way analysis of variance (ANOVA) followed by Tukey’s multiple comparison post hoc test. A *P* value of < 0.05 was considered significant and noted based on the following hierarchy *P < 0.05, **P < 0.01, ***P < 0.001 and ****P < 0.0001.

## Results

### Cell viability by MTT assay

The viability of SIM-A9 and HMC3 cells treated with DMA was assessed by MTT assay (Supplementary Fig. S1). Increasing concentrations of DMA were used to determine the highest non-cytotoxic concentration of DMA in the presence or absence of 1 µg/ml LPS or 3 μM Aβ42. The highest concentration causing less than 20% cell death in SIM-A9 cells was 10 mM. Any change in cell viability at this concentration of DMA was not statistically significant. As for HMC3 cells, the highest concentration of DMA causing less than 20% cell death was 20 mM in unstimulated cells and cells induced by Aβ42 and 30 mM in LPS-stimulated cells. There was no significant change in cell viability at this concentration of DMA. To be consistent with the results in SIM-A9 cells, 10 mM DMA was the highest concentration used in both microglial cell lines in all further experiments.

### DMA inhibits secretion of NO in SIM-A9 cells

**Figure 1:**
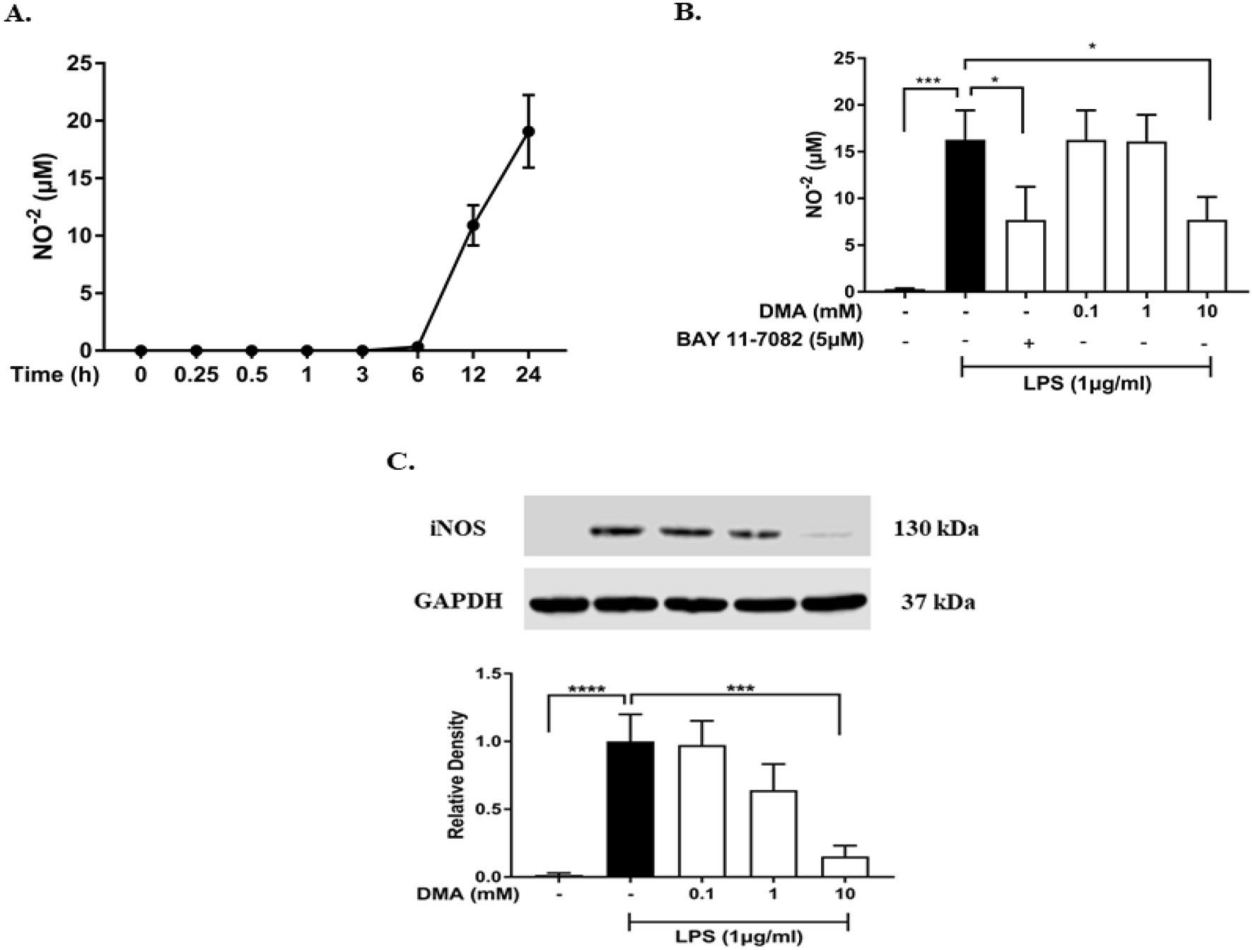
DMA inhibits NO secretion and iNOS expression in SIM-A9 cells induced by LPS. (**A**) Time-course of NO secretion (reflected by NO^−2^ measurements) determined by Griess assay in SIM-A9 cells after incubation with 1 µg/ml LPS. (**B**) NO secretion (reflected by NO^−2^ measurements) in LPS-induced SIM-A9 cells after incubation with various concentrations of DMA. (**C**) Western blot analysis of iNOS expression after incubation with various concentrations of DMA. BAY 11-7082 (5 mM) is included in (**B**) as a positive control. Data shown are means of three independent experiments performed in duplicate. To conserve reagents, membranes were cropped after proteins were transferred and portions of the membrane containing the proteins of interest were used for western blotting. *P < 0.05, ***P < 0.001, ****P < 0.0001.

NO is an important mediator produced by inflammatory cells in response to inflammatory stimuli through the induction of inducible nitric oxide synthase (iNOS)^48^. NO^-2^ levels, an indirect measure of NO production, were significantly increased in SIM-A9 cell supernatants after incubation with 1 µg/ml LPS for 24 h based on a time-course study performed prior to the experiment (Fig. 1A). In the experiment to test DMA’s effect on NO production, 1 µg/ml LPS significantly increased the secretion of NO (reflected by NO^-2^ measurements) from SIM-A9 cells as compared to untreated cells, as expected (*P* < 0.001) (Fig. 1B). DMA at a concentration of 10 mM significantly inhibited the secretion of NO (reflected by decreased NO^−2^ levels^)^ compared to the LPS control (*P* < 0.05). The NF-κB inhibitor BAY 11-7082 (5 µM), used as a positive control, had a similar effect (*P* < 0.05).

Next, the effect of DMA treatment on LPS-induced expression of iNOS in whole cell lysates of SIM-A9 cells was investigated. As expected, there was a significant increase in iNOS expression after a 24 h incubation with 1 µg/ml LPS (*P* < 0.0001) (Fig. 1C). DMA at 10 mM significantly diminished iNOS production (*P* < 0.001).

### DMA regulates ROS production in SIM-A9 cells

**Figure 2:**
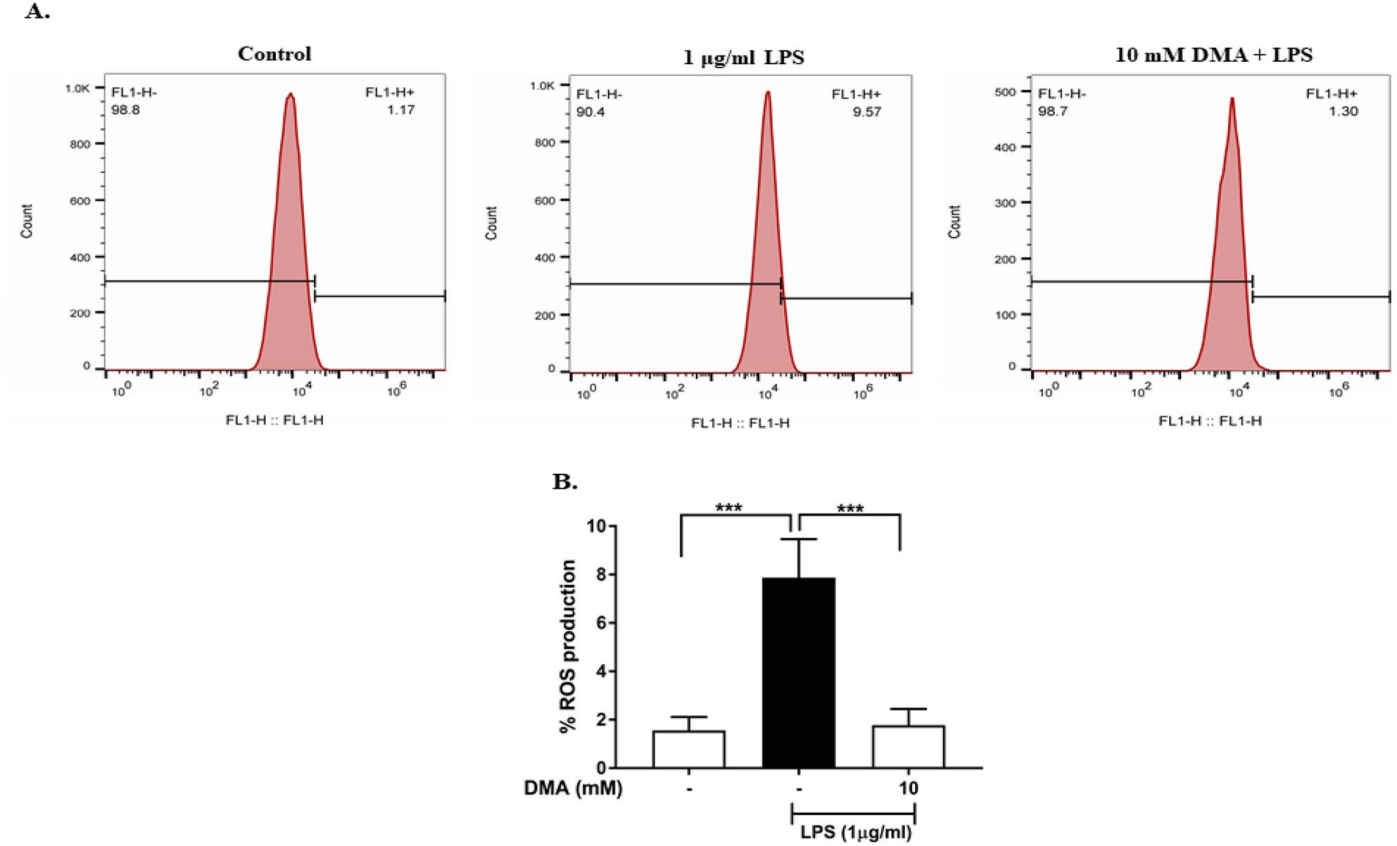
DMA attenuates ROS production in LPS-stimulated SIM-A9 cells. (**A**) Flow cytometry assay of ROS production in SIM-A9 cells induced by LPS in the absence or presence of 10 mM DMA. (**B**) Statistical analysis of ROS production. Data shown are means of three independent experiments performed in duplicate. ***P < 0.001.

ROS serve as critical inflammatory mediators in neuroinflammation and have been implicated in the pathogenesis of AD^8^. Flow cytometry was used to investigate the effect of DMA on ROS production (Fig. 2). ROS were detected in 1.17% of unstimulated SIM-A9 cells (Fig. 2). Not surprisingly, 1 µg/ml LPS significantly increased the production of ROS to 9.57% as compared to the control (*P* < 0.001) (Fig. 2). DMA at a concentration of 10 mM significantly decreased ROS production to 1.30% when compared to the LPS control (*P* < 0.001) (Fig. 2).

### DMA attenuates secretion of cytokines and chemokines in SIM-A9 and HMC3 cells

**Figure 3:**
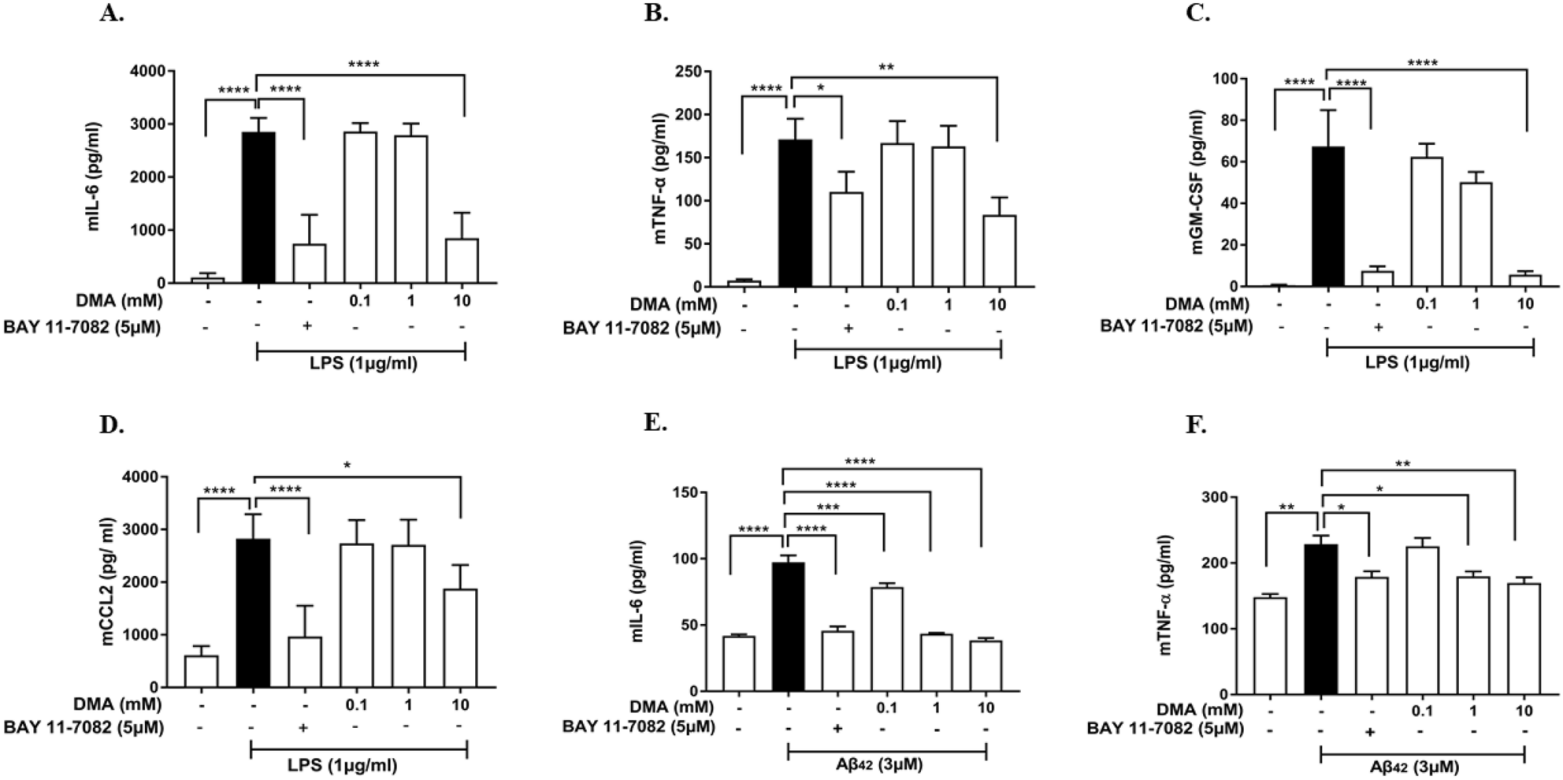
DMA inhibits secretion of cytokines and chemokines in LPS- and Aβ42-induced SIM-A9 cells. Concentrations of (**A**) IL-6, (**B**) TNF-α, (**C**) GM-CSF and (**D**) CCL2 in SIM-A9 cell culture supernatants determined by ELISA after stimulation with 1 µg/ml LPS and (**E**) IL-6 and (**F**) TNF-α after stimulation with 3 μM Aβ42 for 24 h in the absence or presence of various concentrations of DMA. BAY 11-7082 (5 mM) is included as a positive control. Data shown are means of three independent experiments performed in duplicate. *P < 0.05, **P < 0.01, ****P < 0.0001.

ELISA was used to determine the levels of pro-inflammatory cytokines and chemokines in SIM-A9 and HMC3 cell culture supernatants. As expected, LPS (1 µg/ml) significantly increased the secretion of several cytokines, including IL-6, TNF-α and GM-CSF, and chemokine CCL2 in SIM-A9 cells when compared to unstimulated cells (*P* < 0.0001) (Fig. 3A–D). DMA (10 mM) decreased IL-6 production by 70% compared to the LPS control (*P* < 0.0001) (Fig. 3A). Similarly, 10 mM DMA significantly suppressed the increase in TNF-α level induced by LPS (*P* < 0.01) (Fig. 3B). DMA (0.1–10 mM) inhibited GM-CSF secretion induced by LPS in a concentration-dependent manner, with statistical significance at 10 mM DMA (*P* < 0.0001) (Fig. 3C). Similarly, a significant decrease in LPS-induced CCL2 level was found with 10 mM DMA as compared to the LPS control (*P* < 0.05) (Fig. 3D).

The secretion of IL-6 and TNF-α were significantly upregulated by Aβ42 (3 μM) in SIM-A9 cells as compared to unstimulated cells (*P* < 0.0001*, P* < 0.01) (Fig. 3E,F). DMA (0.1–10 mM) significantly inhibited the expression of IL-6 in a concentration-dependent manner as compared to the Aβ42 control (*P* < 0.001, *P* < 0.0001*, P* < 0.0001, respectively) and 10 mM DMA brought IL-6 levels back to baseline (Fig. 3E). DMA (0.1–10 mM) also reduced TNF-α levels in a concentration-dependent fashion, with both 1 mM and 10 mM DMA causing a significant decrease as compared to the Aβ42 control (*P* < 0.05,* P* < 0.01, respectively) (Fig. 3F). BAY 11-7082 (5 μM), used as a positive control, had a similar effect as 10 mM DMA.

**Figure 4:**
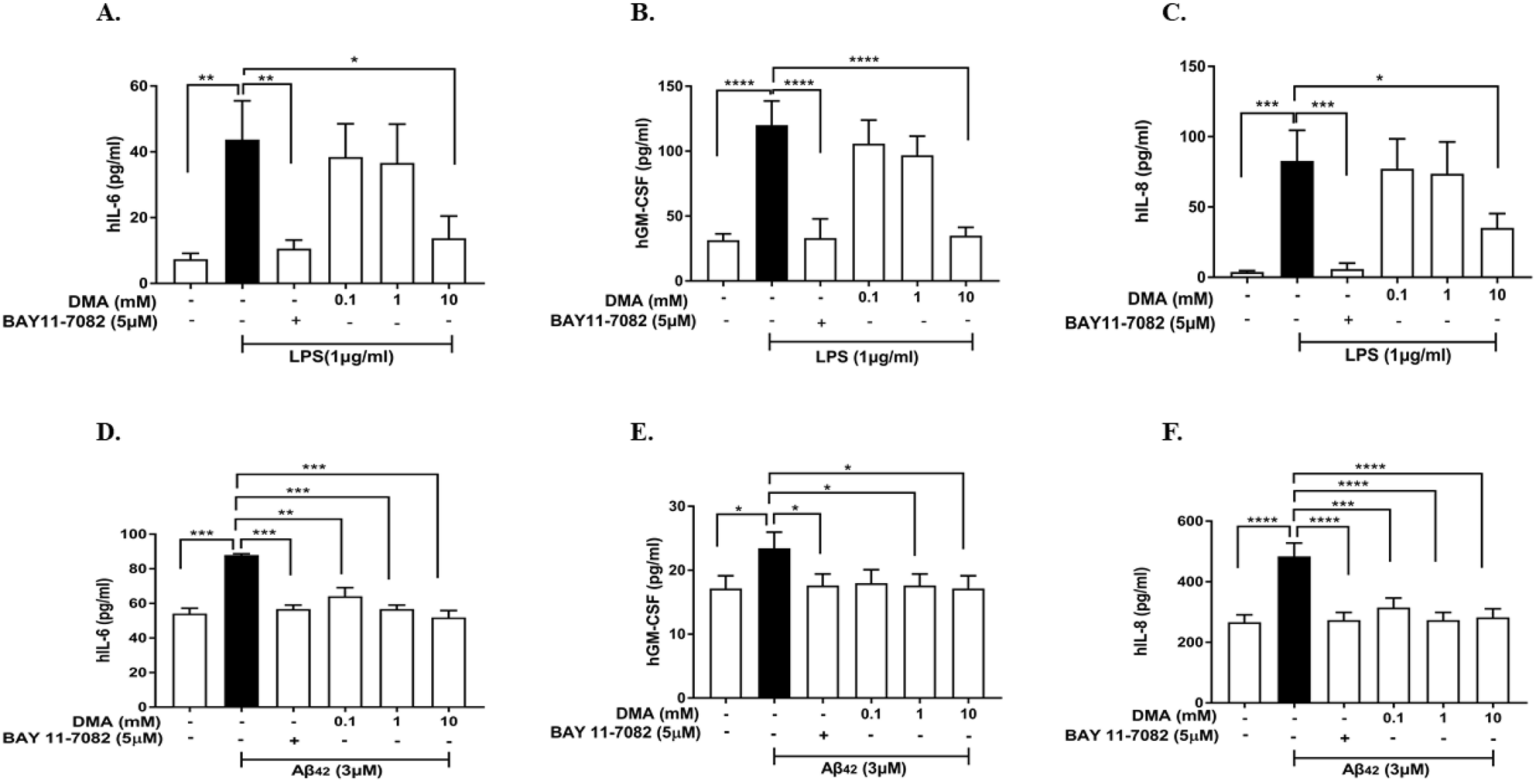
DMA inhibits secretion of cytokines and chemokines in LPS- and Aβ42-induced HMC3 cells. Concentrations of (**A**) IL-6, (**B**) GM-CSF and (**C**) IL-8 in HMC3 cell culture supernatants determined by ELISA after stimulation with 1 µg/ml LPS or 3 μM Aβ42 (**D**–**F**) for 24 h in the absence or presence of various concentrations of DMA. BAY 11-7082 (5 mM) is included as a positive control. Data shown are means of three independent experiments performed in duplicate. *P < 0.05, **P < 0.01, ***P < 0.001, ****P < 0.0001.

LPS (1 µg/ml) was also observed to significantly stimulate cytokine (IL-6 and GM-CSF) and chemokine (IL-8) production in HMC3 cells when compared to untreated cells (*P* < 0.01, *P* < 0.001, *P* < 0.0001) (Fig. 4A–C). Both 0.1 mM and 1 mM DMA slightly decreased the secretion of IL-6 (Fig. 4A), GM-CSF (Fig. 4B) and IL-8 (Fig. 4C), and 10 mM DMA significantly inhibited production of IL-6 (*P* < 0.05), GM-CSF (*P* < 0.0001) and IL-8 (*P* < 0.05) as compared to the LPS control.

The same cytokines and chemokines were evaluated by ELISA in Aβ42-induced HMC3 cells (Fig. 4D–F). Compared to untreated cells, 3 μM Aβ42 was found to significantly induce the production of IL-6 (*P* < 0.001) (Fig. 4D), GM-CSF (*P* < 0.0001) (Fig. 4E) and IL-8 (*P* < 0.05) (Fig. 4F). DMA at all concentrations tested (0.1, 1 mM and 10 mM) significantly downregulated IL-6 (Fig. 4D) and IL-8 (Fig. 4F) levels as compared to the Aβ42 control and 1 mM and 10 mM DMA significantly lowered Aβ42-induced GM-CSF levels (*P* < 0.05) (Fig. 4E). BAY 11-7082 (5 mM) was included as a positive control.

### DMA reduces Aβ42, APP and APP gene expression in SIM-A9 and HMC3 Cells

**Figure 5:**
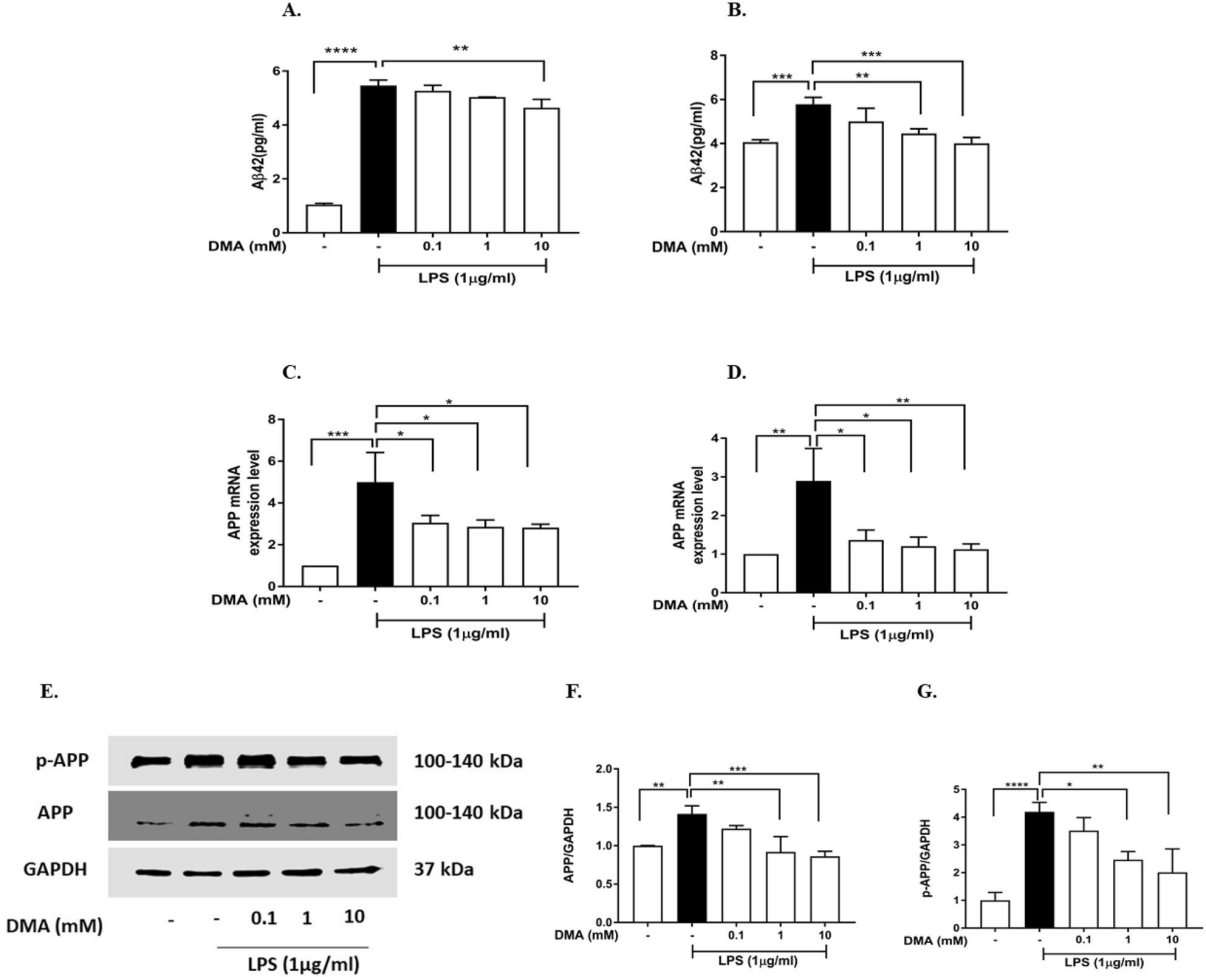
DMA reduces Aβ42, APP and APP gene expression in SIM-A9 cells and HMC3 cells. Aβ42 levels in lysates from (**A**) SIM-A9 cells and (**B**) HMC3 cells determined by ELISA after stimulation with 1 µg/ml LPS for 24 h in the absence or presence of various concentrations of DMA. APP mRNA expression in (**C**) SIM-A9 cells and (**D**) HMC3 cells determined by quantitative RT-PCR after stimulation with 1 µg/ml LPS for 12 h in the absence or presence of various concentrations of DMA. (**E**–**G**) Levels of native and phosphorylated forms of APP in HMC3 lysates determined by western blotting after stimulation with 1 µg/ml LPS for 24 h in the absence or presence of various concentrations of DMA. GAPDH was used as a gel loading control. Data shown are means of three independent experiments*.* To conserve reagents, membranes were cropped after proteins were transferred and portions of the membrane containing the proteins of interest were used for western blotting. **P < 0.01, ***P < 0.001, ****P < 0.0001.

Aβ42 protein levels were significantly upregulated by 1 µg/ml LPS in both SIM-A9 cells (Fig. 5A) and HMC3 cells (Fig. 5B) as compared to untreated cells. DMA (0.1–10 mM) decreased Aβ42 in a concentration-dependent manner, with 10 mM DMA producing a significant decrease when compared to LPS control SIM-A9 cells (*P* < 0.001) (Fig. 5A) and HMC3 cells (*P* < 0.001) (Fig. 5B). Quantitative RT-PCR and western blotting were performed to determine the effect of DMA on APP gene and APP protein expression, respectively. As shown in Fig. 5, 1 µg/ml LPS increased APP mRNA expression in both SIM-A9 cells (*P* < 0.001) (Fig. 5C) and HMC3 cells (*P* < 0.01) (Fig. 5D). All concentrations of DMA tested (0.1, 1 mM and 10 mM) significantly inhibited LPS-induced APP gene expression in SIM-A9 cells (Fig. 5C) and HMC3 cells (Fig. 5D). Native APP and its phosphorylated form (p-APP, phosphorylated at the Threonine (Thr) 668 site) were increased by 1 µg/ml LPS in HMC3 cells (Fig. 5E–G). Both APP and p-APP protein levels were significantly reduced by 1 mM and 10 mM DMA (Fig. 5F,G).

### DMA inhibits degradation of IκBα and nuclear translocation of NF-κB p65 in SIM-A9 and HMC3 cells

**Figure 6:**
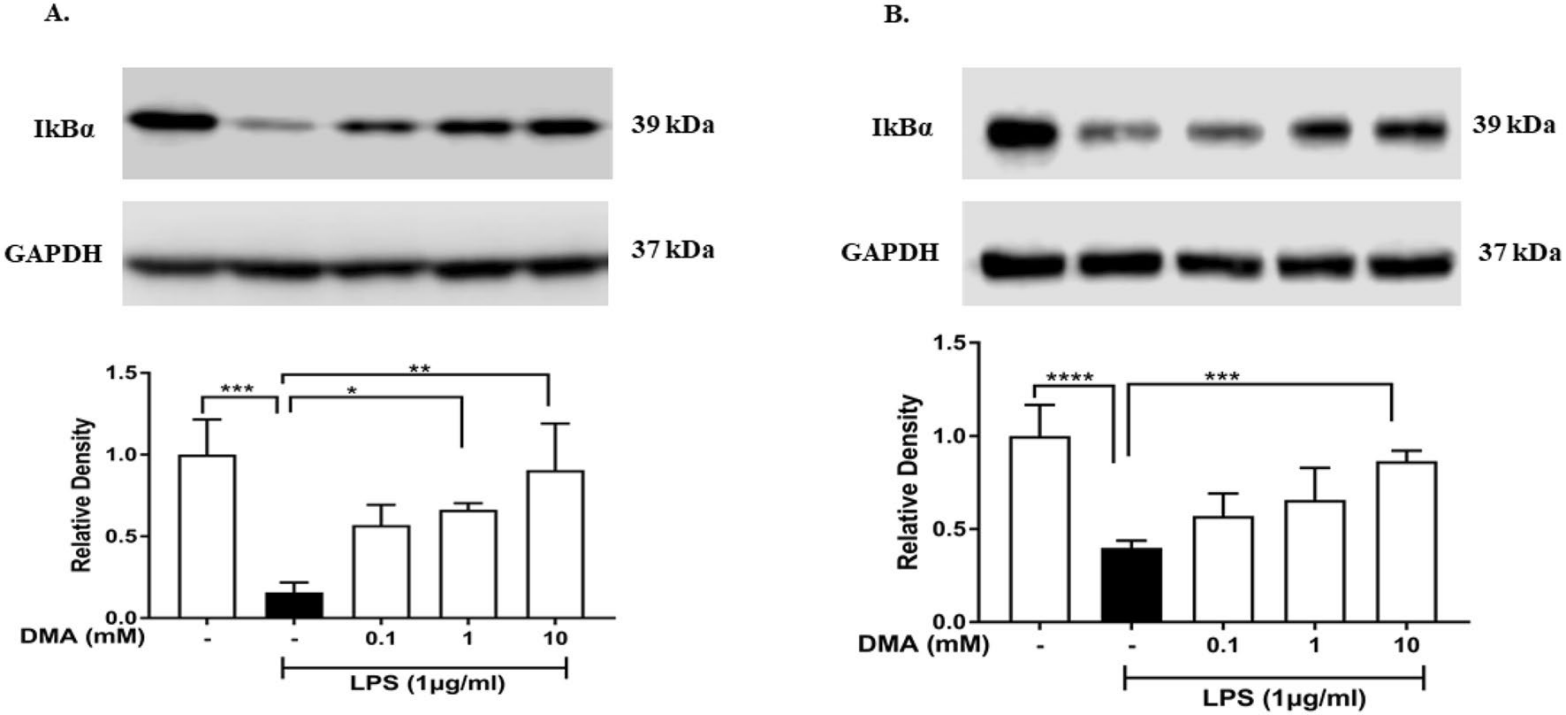
DMA inhibits degradation of IκBα in LPS-stimulated SIM-A9 and HMC3 cells. (**A**) Levels of IκBα determined by western blotting in SIM-A9 cells after stimulation with 1 µg/ml LPS for 15 min in the absence or presence of various concentrations of DMA. (**B**) Levels of IκBα determined by western blotting in HMC3 cells after stimulation with 1 µg/ml LPS for 30 min in the absence or presence of various concentrations of DMA. GAPDH was used as a gel loading control. Data shown are means of three independent experiments. To conserve reagents, membranes were cropped after proteins were transferred and portions of the membrane containing the proteins of interest were used for western blotting. *P < 0.05, **P < 0.01, ***P < 0.001, ****P < 0.0001.

We have previously shown that DMA prevents activation of the NF-κB pathway by inhibiting IκBα degradation and nuclear translocation of the p65 subunit of NF-κB in LPS-stimulated RAW 264.7 mouse macrophage cells^29,36^. Therefore, we hypothesized that DMA would have the same mechanism of action in microglial cells. IκBα was degraded 15 min after exposure to 1 µg/ml LPS in SIM-A9 cells (Fig. 6A) and HMC3 cells (Fig. 6B) as compared to untreated cells (*P* < 0.001). DMA prevented LPS-induced IκBα degradation in a concentration-dependent manner, with both 1 mM and 10 mM DMA significantly increasing IκBα levels as compared to the LPS control in SIM-A9 cells (*P* < 0.05, *P* < 0.01, respectively, Fig. 6A) and 10 mM DMA significantly increasing IκBα levels as compared to the LPS control in HMC3 cells (*P* < 0.001, Fig. 6B).

**Figure 7:**
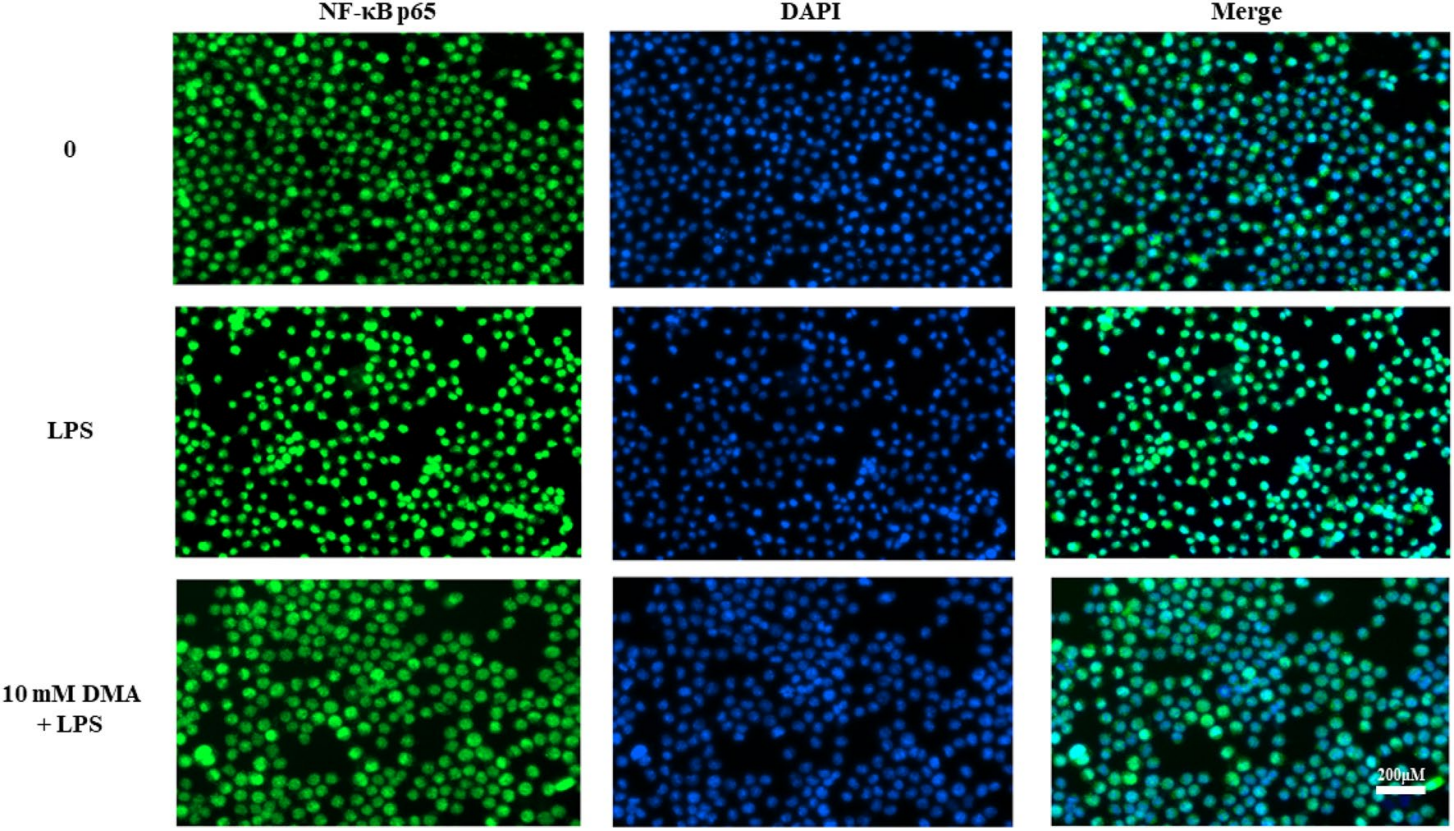
DMA inhibits nuclear translocation of NF-κB p65 in LPS-stimulated SIM-A9 cells. SIM-A9 cells were incubated with or without 10 mM DMA in the absence or presence of LPS (1 µg/ml) for 15 min. NF-κB p65 was stained green and nuclei were stained blue with DAPI**.** Scale bars: 200 μm.

DMA’s effect on nuclear translocation of the p65 unit of NF-κB in LPS-stimulated microglial cells was then tested. In Fig. 7, SIM-A9 cells were incubated with anti-NF-κB p65 antibodies tagged with green fluorescence and a DAPI stain was used to display the nuclei in blue. Immunoreactivity for the p65 unit in unstimulated SIM-A9 cells was localized primarily in the cytoplasm, as indicated by the green fluorescence surrounding but not overlapping the nucleus (blue) (Fig. 7, first row). However, a 15-min exposure to LPS led to translocation of the NF-κB p65 subunit into the nucleus, reflected by overlap of the green and blue stains (Fig. 7, second row). LPS-stimulated cells treated with 10 mM DMA displayed significantly reduced nuclear staining for p65 compared to the LPS control, consistent with DMA’s ability to inhibit LPS-induced nuclear translocation of NF-κB (Fig. 7, third row).

**Figure 8:**
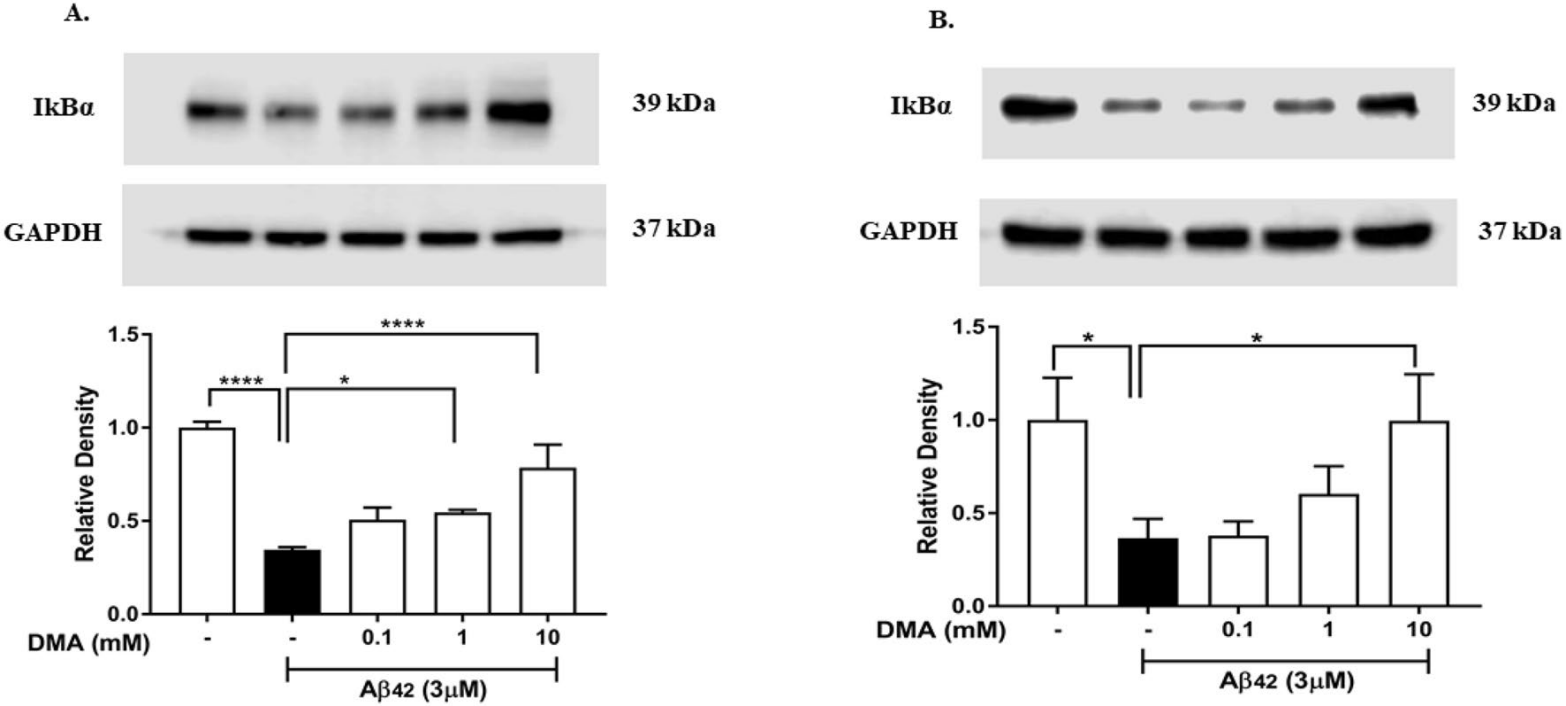
DMA inhibits degradation of IκBα in Aβ42-stimulated SIM-A9 and HMC3 cells. (**A**) Levels of IκBα determined by western blotting in SIM-A9 cells after stimulation with 3 mM Aβ42 for 1 h in the absence or presence of various concentrations of DMA. (**B**) Levels of IκBα determined by western blotting in HMC3 cells after stimulation with 3 mM Aβ42 for 1 h in the absence or presence of various concentrations of DMA. GAPDH was used as a gel loading. To conserve reagents, membranes were cropped after proteins were transferred and portions of the membrane containing the proteins of interest were used for western blotting. *P < 0.05, ****P < 0.0001.

To determine the mechanism whereby DMA suppresses the secretion of inflammatory mediators induced by Aβ42, we tested DMA’s effect on IκBα degradation in Aβ42-stimulated microglial cells. Figure 8 shows that 3 μM Aβ42 significantly induced degradation of IκBα in SIM-A9 cells (*P* < 0.0001) (Fig. 8A, Lane 2) and HMC3 cells (*P* < 0.05) (Fig. 8B, Lane 2). The degradation of IκBα was attenuated by DMA in a concentration-dependent fashion, with 10 mM DMA showing significant attenuation of IκBα loss in both SIM-A9 cells (*P* < 0.0001) (Fig. 8A, Lane 5) and HMC3 cells (*P* < 0.05) (Fig. 8B, Lane 5).

**Figure 9:**
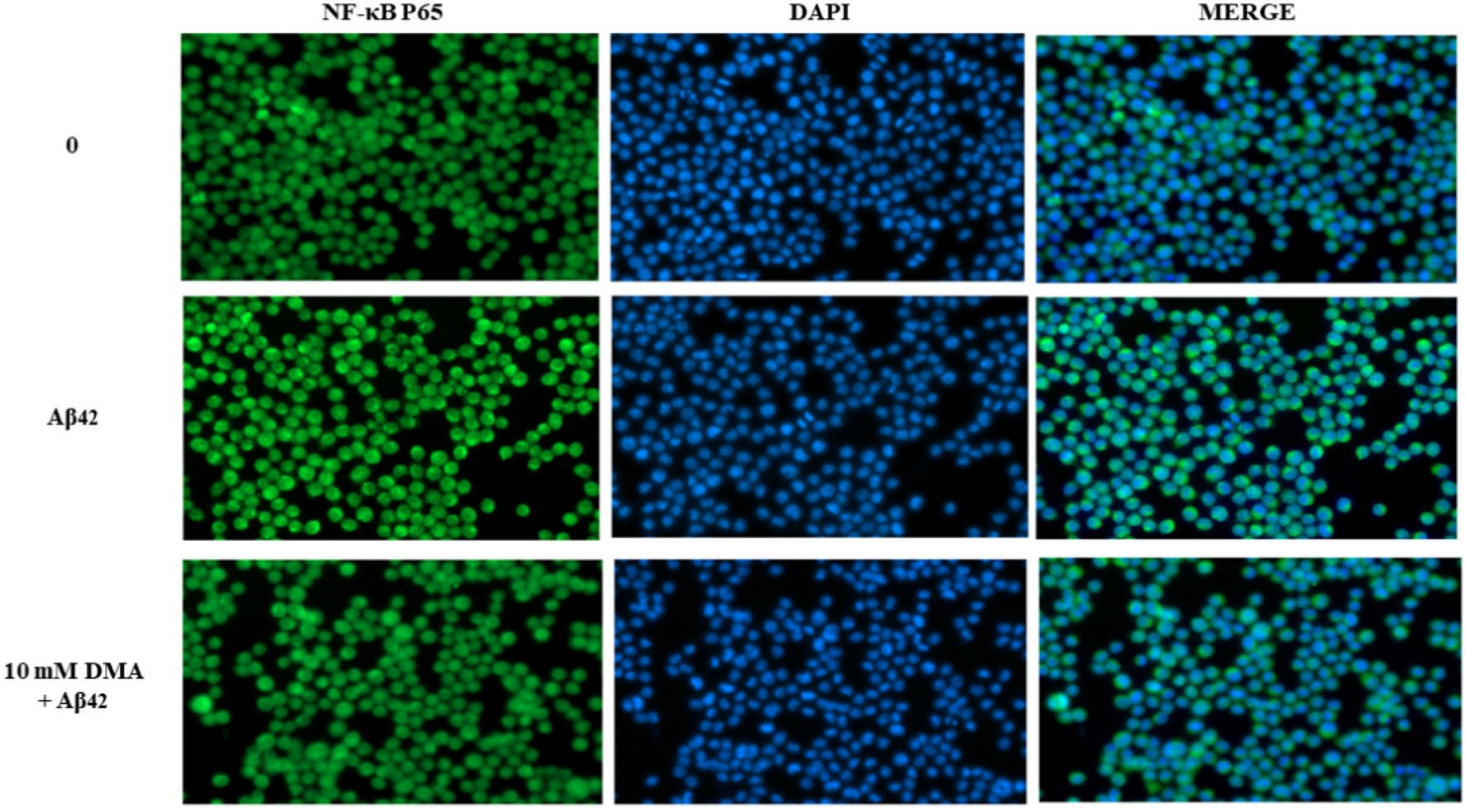
DMA inhibits nuclear translocation of NF-κB p65 in Aβ42-stimulated SIM-A9 cells. SIM-A9 cells were incubated with or without 10 mM DMA in the absence or presence of Aβ42 (3 mM) for 1 h. NF-κB p65 was stained green and nuclei were stained blue with DAPI. Scale bars: 200 μm.

DMA’s effect on NF-κB nuclear translocation in Aβ42-stimulated cells was also tested. As expected, immunoreactivity for the p65 unit in untreated SIM-A9 cells was localized primarily in the cytoplasm (Fig. 9). However, a 1 h exposure to 3 mM Aβ42 led to translocation of NF-κB into the nucleus, reflected by overlapping green and blue stains. Aβ42-induced cells treated with 10 mM DMA displayed significantly reduced nuclear staining in comparison with the Aβ42 control, consistent with DMA’s ability to prevent Aβ42-induced nuclear translocation of NF-κB p65 (Fig. 9).

### DMA does not affect levels of mitogen-activated protein kinase (MAPK) signaling pathway proteins in SIM-A9 cells

**Figure 10:**
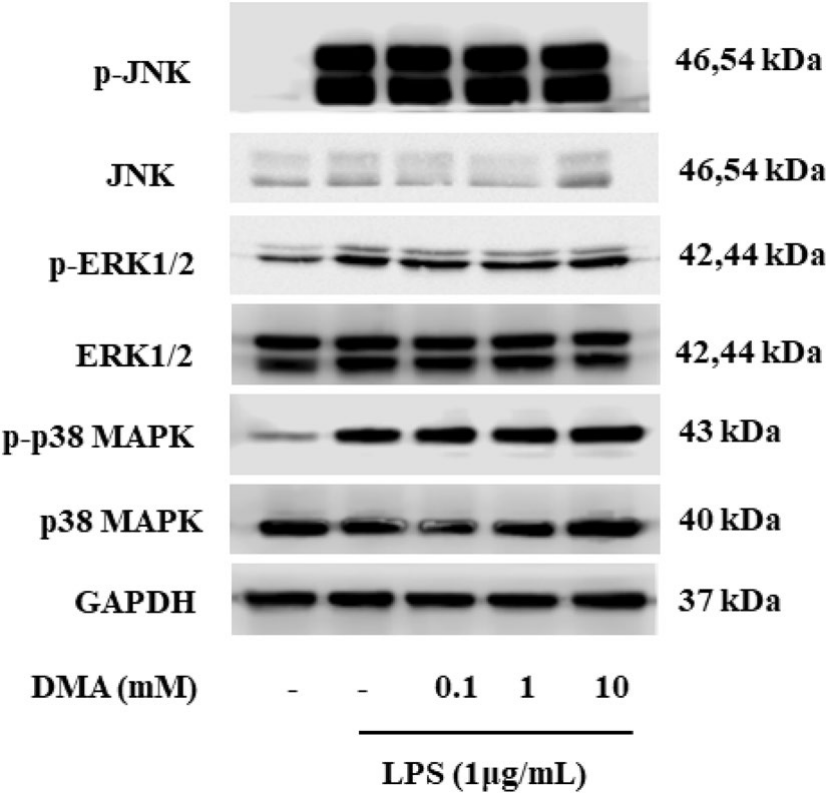
DMA does not affect levels of MAPK signaling pathway proteins in SIM-A9 cells. Immunoblots of MAPK signaling pathway proteins in SIM-A9 cells after stimulation with LPS (1 µg/ml) for 15 min in the absence or presence or various concentrations of DMA. GAPDH was used as a gel loading control. To conserve reagents, membranes were cropped after proteins were transferred and portions of the membrane containing the proteins of interest were used for western blotting.

The expression of native and phosphorylated forms of JNK, ERK1/2 and p38 in SIM-A9 cells in the absence or presence of stimulation with LPS (1 µg/ml) for 15 min and varying concentrations of DMA were evaluated by immunoblotting (Fig. 10). Phosphorylated JNK, ERK1/2 and p38 MAPK (p-JNK, p-ERK1/2 and p-p38 MAPK) were increased with LPS stimulation (Fig. 10, Lane 2). However, DMA (0.1–10 mM) did not significantly change expression levels of p-JNK, p-ERK1/2 or p-p38 MAPK as compared to LPS controls (Fig. 10, Lanes 3–5). DMA also had no significant effect on the native forms of JNK, ERK1/2 and p38 MAPK (Fig. 10, Lane 3–5).

### DMA inhibits secretion of pro-inflammatory cytokines and chemokines from homogenized rat hippocampal slices

**Figure 11:**
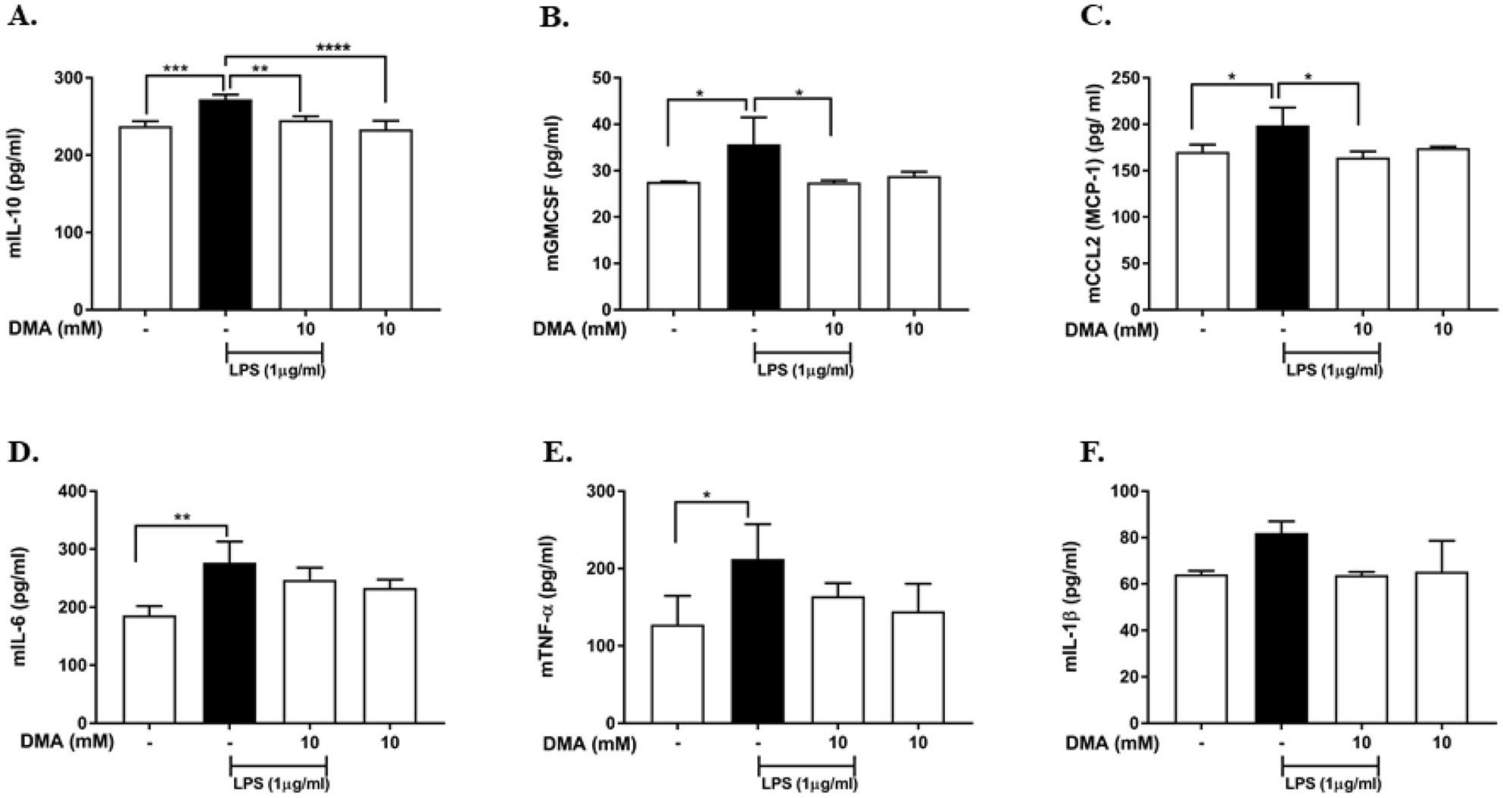
DMA inhibits secretion of cytokines and chemokines in LPS-induced rat hippocampal slice homogenates. ELISA was performed to determine the concentration of (**A**) IL-10, (**B**) GM-CSF, (**C**) CCL2, (**D**) IL-6, (**E**) TNF-α and (**F**) IL-1β in rat hippocampal slice homogenates after stimulation with 1 µg/ml LPS for 15 h in the absence or presence or various concentrations of DMA. Data shown are means of three independent experiments performed in duplicate. *P < 0.05, **P < 0.01, ***P < 0.001, ****P < 0.0001.

Ex vivo cultures provide a platform to study mixed populations of cells while maintaining the three-dimensional structure of the tissue. The hippocampus was chosen because memory loss is a prominent feature of neurodegenerative disorders. Based on LDH assay, 10 mM DMA produced no significant loss of hippocampal cell viability in the absence or presence of LPS (Supplementary Fig. S2A) or Aβ42 (Supplementary Fig. S2B). Thus, 10 mM DMA was used in ex-vivo experiments. LPS (1 µg/ml) significantly increased the production of IL-10, GM-CSF, CCL2, IL-6 and TNF-α (Fig. 11A–D) and produced a non-significant increase in IL-1β (Fig. 11E) in hippocampal explants when compared to untreated controls. DMA (10 mM) significantly decreased IL-10, GM-CSF and CCL2 levels as compared to LPS controls (Fig. 11A–C). DMA (10 mM) also inhibited the secretion of IL-6, TNF-α and IL-1β; however, no significant differences were found (Fig. 11D–F).

**Figure 12:**
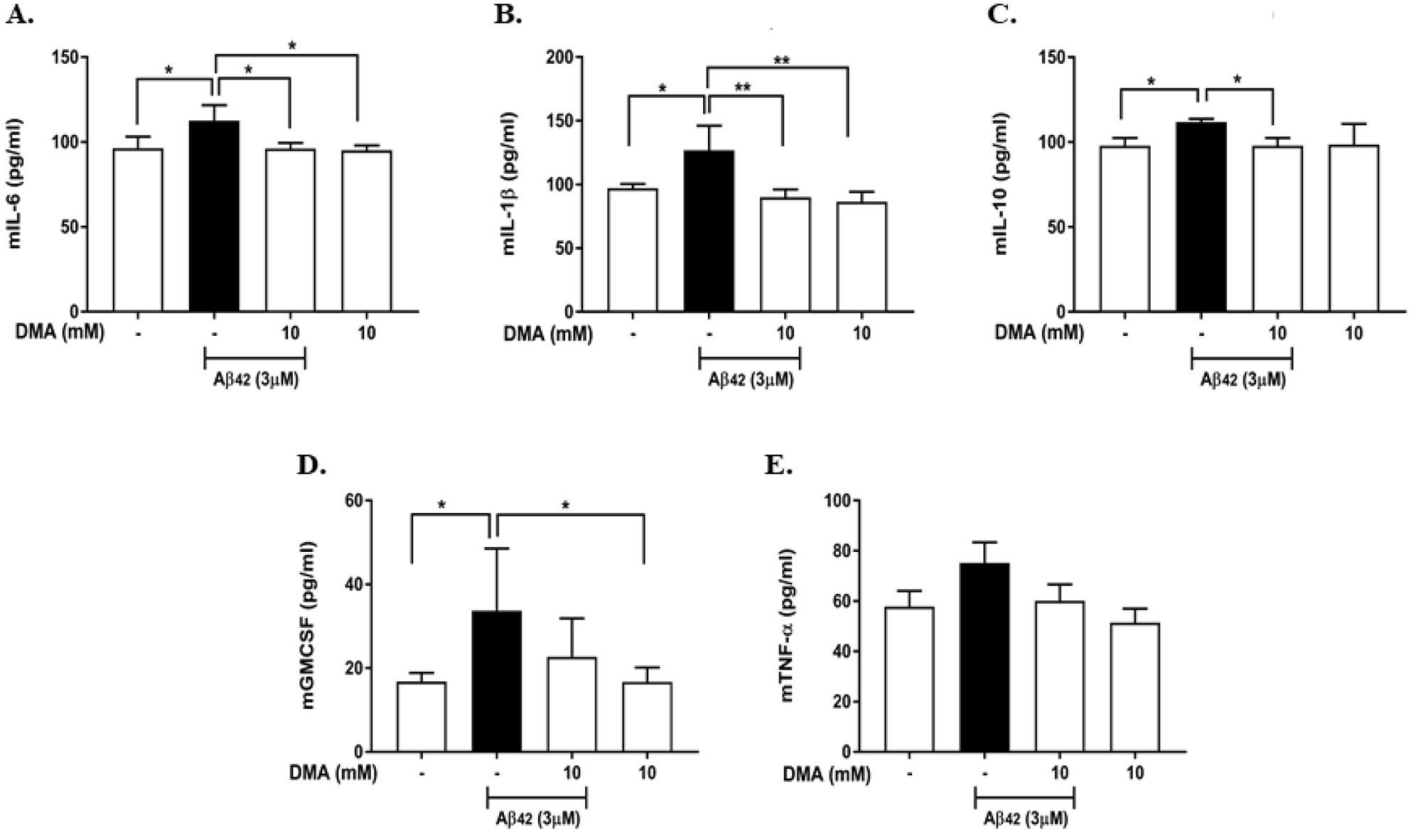
DMA suppresses secretion of cytokines in Aβ42-induced rat hippocampal slice homogenates. ELISA was performed to determine the effect of DMA on levels of (**A**) IL-6, (**B**) IL-1β, (**C**) IL-10, (**D**) GM-CSF and (**E**) TNF-α in rat hippocampal slice homogenates after stimulation with 3 μM Aβ42 for 15 h in the absence or presence or various concentrations of DMA. Data shown are means of three independent experiments performed in duplicate. *P < 0.05, **P < 0.01.

Aβ42 (3 μM) significantly increased the production of IL-6, IL-1β, IL-10 and GM-CSF (Fig. 12A–D) and produced a non-significant increase in the production of TNF-α (Fig. 12E). DMA (10 mM) significantly inhibited the production of IL-6, IL-1β and IL-10 as compared to the LPS controls (Fig. 12A–C) and reduced GM-CSF and TNF-α levels, although not significantly (Fig. 12D,E).

## Discussion

AD is the most common form of dementia^49^, afflicting over 6 million Americans in 2020 and predicted to affect 14 million people by 2060 due to aging of the baby boomer generation and increasing members of the oldest old^50,51^. Despite over a century of investigation, its pathogenesis remains incompletely understood^52^. Currently, the FDA has approved four prescription drugs (donepezil, galantamine, rivastigmine and memantine) to treat the symptoms of different stages of AD. These drugs, however, only provide modest improvement of certain symptoms for limited times and are unable to arrest the progression of AD^53,54^. On June 7^th^, 2021, The FDA approved the first Aβ human monoclonal antibody, Aducanumab (marketed as Aduhelm), which was introduced by Biogen^17,55^. Although this development was initially met with optimism, concerns related to both efficacy and safety have dampened enthusiasm about this new drug^8,56,57^.

In this work we recognize the importance of neuroinflammation and oxidative stress in the complex array of brain dysfunction leading to AD. In our hands, neuroinflammation induced by LPS in vitro and ex vivo triggers aberrant microglial activation causing increased production of inflammatory factors, such as ROS, NO, pro-inflammatory cytokines and chemokines. ROS itself is mainly generated in mitochondria as a metabolic by-product, beneficial for some cellular processes, including activation of transcription factors, protein phosphorylation, cell signaling, apoptosis and immunity^58^. However, increased production of ROS induced by LPS stimulates excessive generation of free radicals, resulting in the imbalance between free radicals and antioxidant defenses which is termed oxidative stress^59^. Oxidative stress is detrimental to cellular structures, causes DNA damage and mitochondrial dysfunction and interferes with normal CNS functions^60–63^. Oxidative stress is also implicated in the pathogenesis of AD, based on the finding of abnormal oxidative stress in AD brains and cerebrospinal fluid^64^. Moreover, a myriad of studies have shown that oxidative stress is involved in Aβ accumulation^65,66^. We report here that DMA significantly diminishes LPS-induced ROS expression in SIM-A9 cells.

Besides functioning as a crucial inflammatory mediator, NO is a well-known free radical in intracellular signaling cascades and affects neuronal activity^67^. NO reacts with another free radical, superoxide anion, to produce peroxynitrite, which is toxic to cells and participates in chronic inflammatory and neurodegenerative functions^67,68^. In our SIM-A9 cells, NO is produced through the induction of iNOS which is triggered by activation of the NF-κB pathway^25^. As expected, not only did it increase NO secretion, but LPS also upregulated the expression of iNOS. DMA (10 mM) significantly downregulated both levels of NO and expression of iNOS.

LPS significantly stimulates the secretion of IL-6, IL-8, IL-1β, TNF-α, CCL2, GM-CSF and IL-10 in both microglia and hippocampal slices. In particular, IL-6, IL-1β and TNF-α are vital pro-inflammatory cytokines involved in the accumulation of Aβ in AD. High levels of these cytokines inhibit the phagocytosis and clearance of Aβ by microglia and lead to increased amounts of Aβ by promoting cleavage of APP in AD brains^33–35,69,70^. It is interesting that IL-10 is up-regulated by LPS and Aβ in cultured microglial and in hippocampal organotypic slices, as IL-10 has both pro-inflammatory and anti-inflammatory properties^71^. DMA significantly attenuated the production of pro-inflammatory cytokines and chemokines induced by LPS in both microglia and hippocampal slices. Furthermore, to mimic the milieu of the AD brain, we stimulated the microglia and hippocampal slices with exogenous Aβ, which resulted in secretion of pro-inflammatory cytokines and chemokines (IL-6, IL-8, IL-1β, TNF-α, CCL2, GM-CSF and IL-10) in both the in-vitro and ex-vivo models. DMA significantly inhibited the generation of these inflammatory mediators. Taken together with the ROS and NO results, we conclude that DMA is a potent anti-neuroinflammatory agent.

There are at least two signaling cascades that may be involved in neuroinflammation: the NF-κB and MAPK signaling pathways^72^. Previously, our laboratory reported that DMA inhibited inflammatory responses via inhibition of the NF-κB signaling pathway but had no effect on the MAPK pathway^29^. Consistent with our previous work, we report here that DMA inhibited the degradation of IκBα and translocation of NF-κB p65 into the nucleus in stimulated cells but had no effect on the MAPK pathway. Besides its effect on neuroinflammation, we also report here that DMA significantly downregulated the gene and protein expression of APP, further contributing to the decreased expression of Aβ in microglia. Interestingly, DMA did not affect levels of tau protein (data not shown).

We recognize that many factors affecting the efficacy of DMA in the CNS may not be recapitulated in our ex-vivo and in-vitro models. For example, stimulation of microglia and hippocampal slices with both LPS and Aβ increased IL-10 levels, whereas IL-10 has anti-inflammatory function in many in-vivo systems. Pre-clinical trials to test DMA’s efficacy in in-vivo models of neuroinflammation and/or AD are planned.

## Conclusion

**Figure 13:**
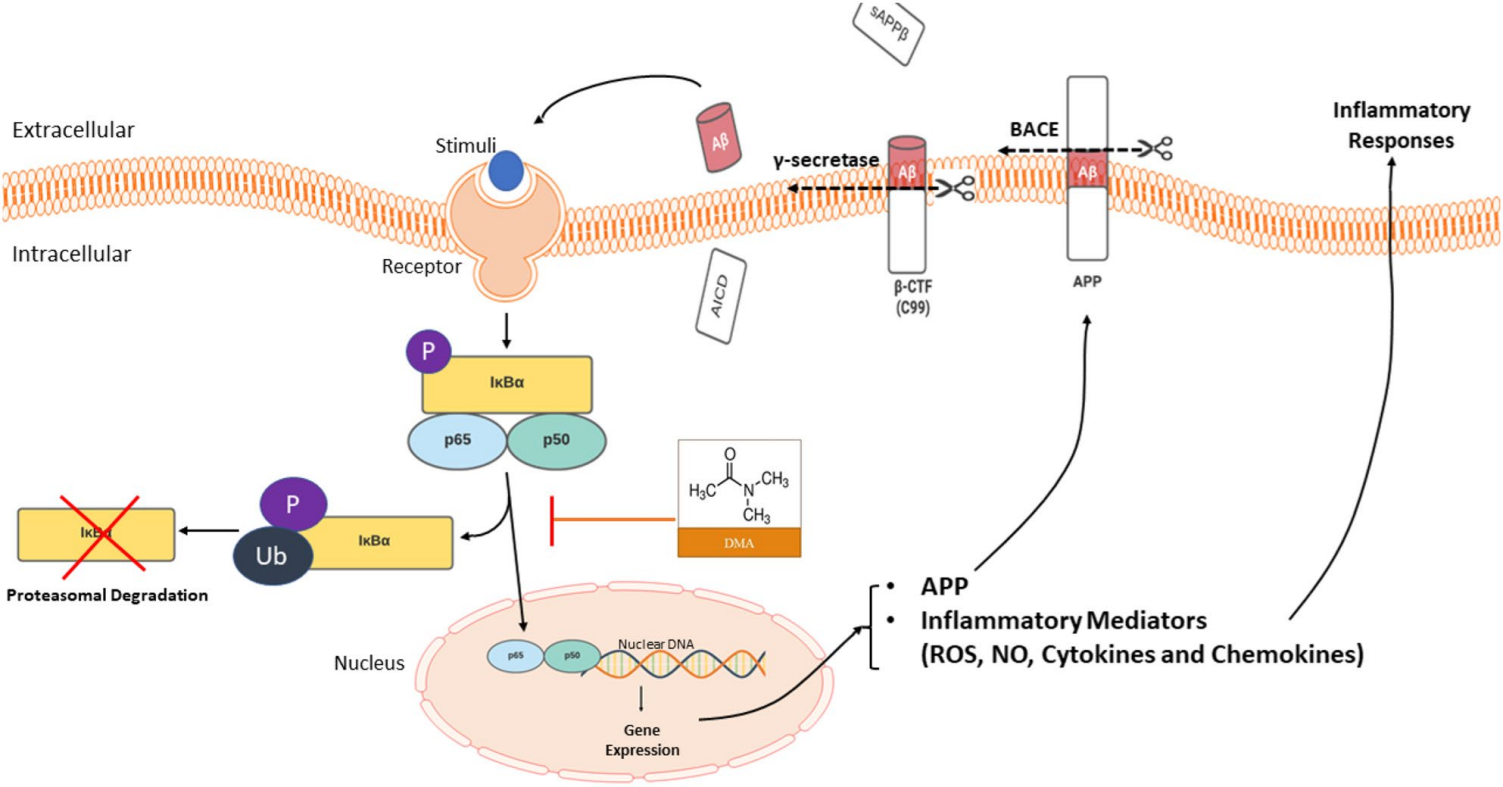
The proposed mechanism of action of DMA in neuroinflammation and AD. Stimulated pattern recognition receptors in microglia activate the NF-κB pathway, leading to degradation of IκBα and translocation of NF-κB p65 into the nucleus to trigger inflammatory responses and upregulation of Aβ. Increased levels of Aβ exacerbate NF- κB pathway activation and inflammation, producing a feed-forward loop. DMA suppresses IκBα degradation and NF-κB p65 nuclear translocation, which attenuates neuroinflammation.

To the best of our knowledge, the present study is the first report of DMA’s effect on neuroinflammation and APP production. A summary of DMA’s proposed mechanism of action is provided in Fig. 13. Briefly, stimulated pattern recognition receptors in microglia activate the NF-κB pathway, leading to degradation of IκBα and translocation of NF-κB p65 into the nucleus. P65 acts as a transcription factor and drives inflammatory responses, upregulation of APP and increased β-amyloid converting enzyme 1 (BACE1) activity. The resulting increase in levels of Aβ exacerbates NF-κB pathway activation and inflammation, producing a feed-forward loop. DMA suppresses IκBα degradation and NF-κB p65 nuclear translocation, dismantling this feed-forward loop, attenuating neuroinflammation and holding new promise for the treatment of AD.

## Supplementary Information

Supplementary Information 1.

Supplementary Information 2.

## Data Availability

Original data are available from the corresponding author upon reasonable request.
