# Supplementary material for: N,N-dimethylacetamide targets neuroinflammation in Alzheimer’s disease in in-vitro and ex-vivo models
Source: Sci Rep. 2023 May 1;13:7077. doi: 10.1038/s41598-023-34355-w (PMC10151369; doi:10.1038/s41598-023-34355-w)
Supplement: Supplementary file 1 — Supplementary Information 1. [file 41598_2023_34355_MOESM1_ESM.pdf]

Supplemental Data

Table S1. The sequence of genes and primer pairs used in RT-PCR

Species	Target Gene	Primer Sequences	
		Forward Primer	Reverse Primer
Human Only	APP	5’-CTGTGGCAGACTGAACATGC-3’	5’-ACCAACTAAGCAGCGGTAGG-3’
	GAPDH	5’-CTGGGCTACACTGAGCACC-3’	5’-AGTGGTCGTTGAGGGCAATG-3’
Mouse Only	APP	5’-TGTGTCCCATTCTTTTACGGC-3’	5’-AACTTTGGGTTGACACGCTG-3’
	GAPDH	5’-GCCCTTGAGCTAGGACTGGATA-3’	5’-GAGGGCTGCAGTCCGTATTT-3’

Table S2. List of primary antibodies used in Western blotting

**Antibody**	**Dilution**	**Catalog**	**Host**	**Manufacturer**
iNOS	1:1,000	#2977	Rabbit	Cell Signaling Tech.
p-APP (Thr668)	1:1,000	#6986	Rabbit	Cell Signaling Tech.
APP	1:1,000	#2452	Rabbit	Cell Signaling Tech.
p-Tau (Thr181)	1:500	#12885	Rabbit	Cell Signaling Tech.
p-Tau (Thr231)	1:500	#71429	Rabbit	Cell Signaling Tech.
Tau 46	1:500	#4019	Mouse	Cell Signaling Tech.
IκBα	1:1,000	#2452	Rabbit	Cell Signaling Tech.
p-p38 MAPK	1:1,000	#4631	Rabbit	Cell Signaling Tech.
p38 MAPK	1:1,000	#9212	Rabbit	Cell Signaling Tech.

p-ERK1/2	1:1,000	#9101	Rabbit	Cell Signaling Tech.
ERK1/2	1:1,000	#9102	Rabbit	Cell Signaling Tech.
p-JNK	1:1,000	#9251	Rabbit	Cell Signaling Tech.
JNK	1:1,000	#3708	Mouse	Cell Signaling Tech.
p-Akt	1:1,000	#9271	Rabbit	Cell Signaling Tech.
Akt	1:1,000	#9272	Rabbit	Cell Signaling Tech.
GSK-3β	1:1,000	#9315	Rabbit	Cell Signaling Tech.
GAPDH	1:1,000	#2118	Rabbit	Cell Signaling Tech.

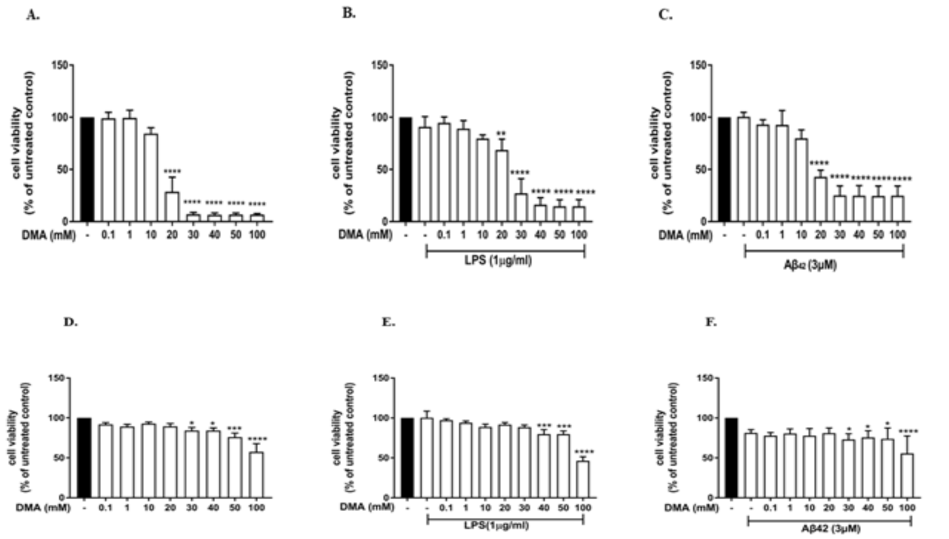

Figure S1. **Viability of SIM-A9 and HMC3 cells treated with DMA.** MTT assay was performed on (A) SIM-A9 cells after incubation with increasing concentrations of DMA, (B) LPS-induced SIM-A9 cells after incubation with increasing concentrations of DMA, (C) Aβ42-induced SIM-A9 cells after incubation with increasing concentrations of DMA, (D) HMC3 cells after incubation with increasing concentrations of DMA, (E) LPS-induced HMC3 cells after incubation with increasing concentrations of DMA. (F) Aβ42-induced HMC3 cells after incubation with increasing concentrations of DMA. Data shown are means of three independent experiments performed in duplicate. **P*<.05, ***P*<.01, ****P*<.001, *****P*<.0001.

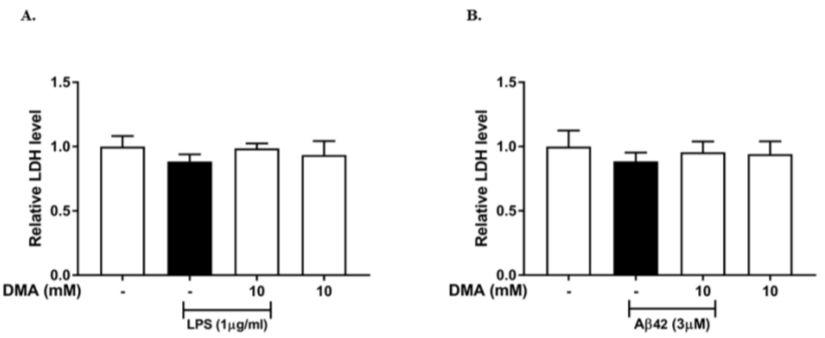

Figure S2. **Viability of rat hippocampal slice homogenates treated with DMA.** LDH assay was performed on hippocampal slices in the absence or presence of (A) 1 μg/ml LPS or (B) 3 μM Aβ42 for 15 h. Data shown are means of three independent experiments.
