# Supplementary material for: N,N-dimethylacetamide targets neuroinflammation in Alzheimer’s disease in in-vitro and ex-vivo models
Source: Sci Rep. 2023 May 1;13:7077. doi: 10.1038/s41598-023-34355-w (PMC10151369; doi:10.1038/s41598-023-34355-w)
Supplement: Supplementary file 2 — Supplementary Information 2. [file 41598_2023_34355_MOESM2_ESM.docx]

Supplemental Information

Original western blots are below.


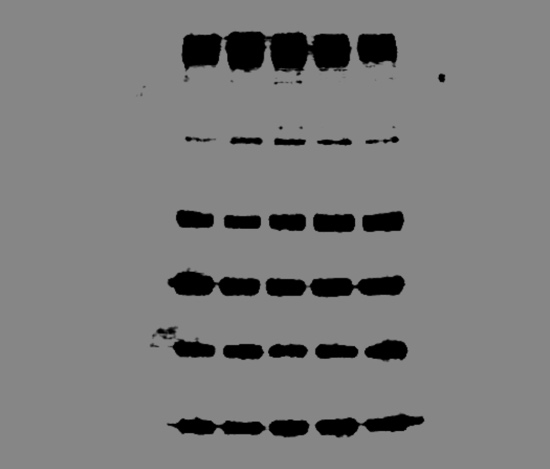

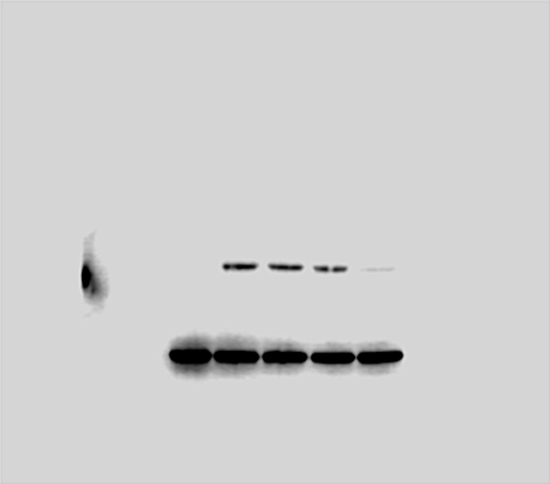


**Figure 1C**. The top row of bands is iNOS; the bottom row of bands is GAPDH.

130 kDa

iNOS

37 kDa

GAPDH


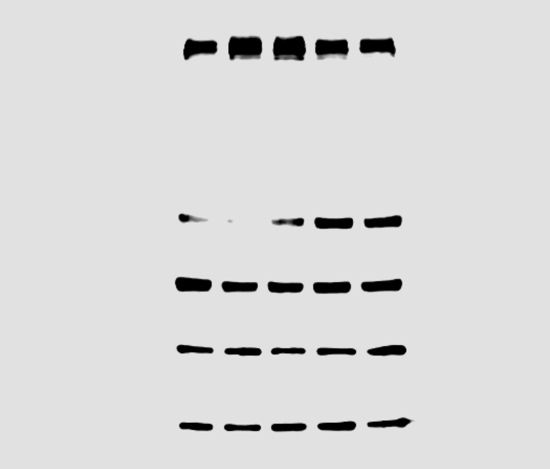


100-140 kDa

pAPP

100-140 kDa

pAPP

100-140 kDa

APP

GAPDH

37 kDa

37 kDa

GAPDH

**Figure 5E**. These are two exposures of the same blot. The top row of bands is p-APP; the second row of bands (only visible in the blot on the left) is APP and the bottom row of bands is GAPDH.


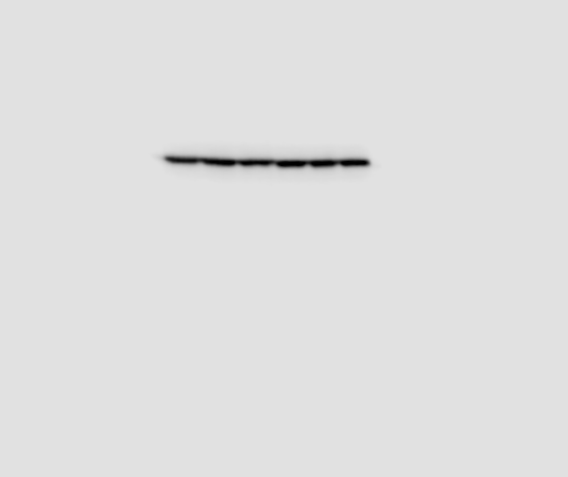

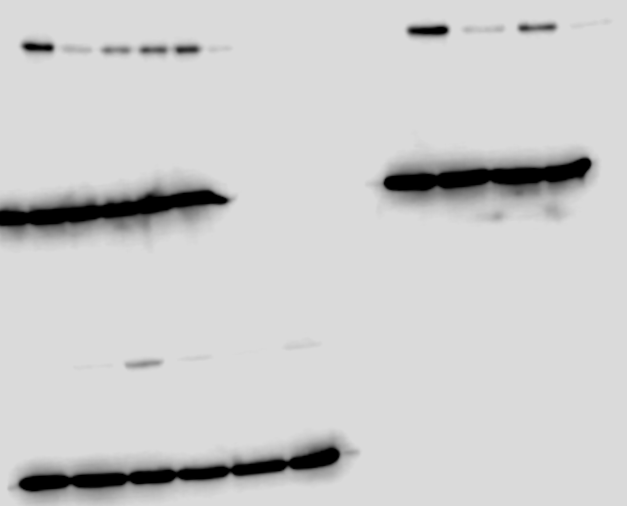


39 kDa

IκBα

GAPDH

37 kDa

**Figure 6A**. The top row of bands indicated on the left is IκBα. A longer exposure time was used in the manuscript figure. The row of bands on the right is GAPDH.


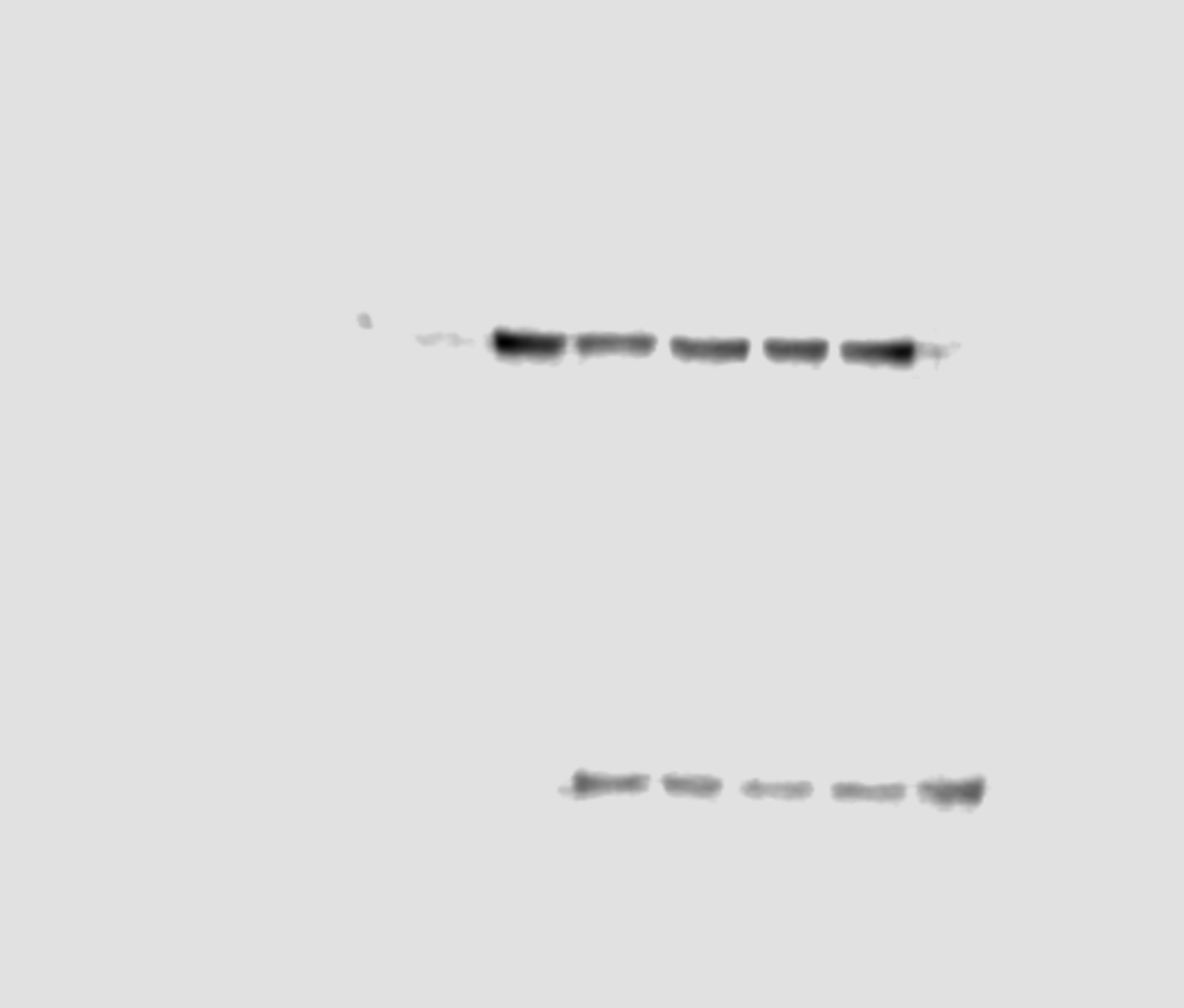

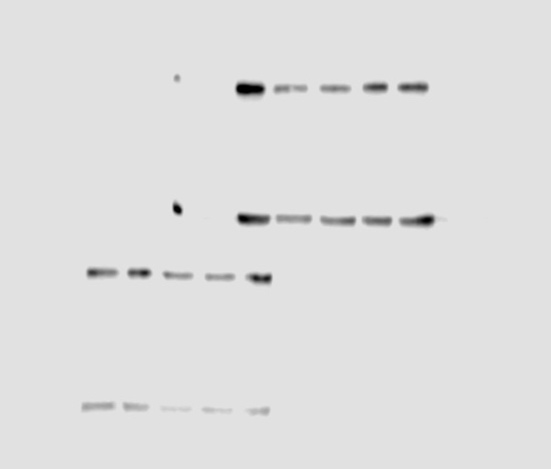


37 kDA

GAPDH

**Figure 6B.** The top row of bands on the left is IκBα. The top row of bands on the right is GAPDH.

IκBα

39 kDa


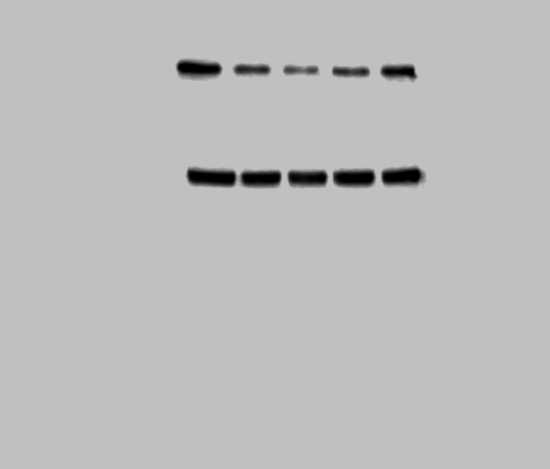

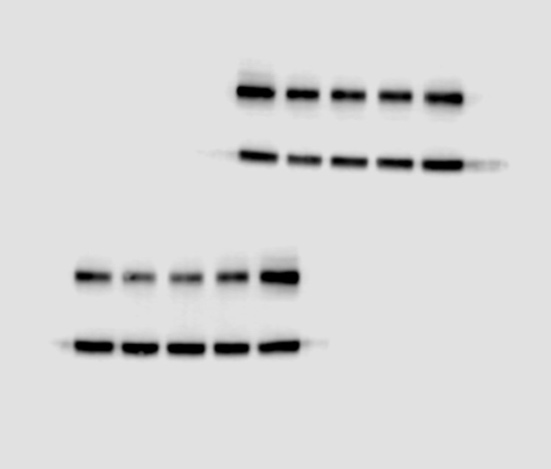


IκBα

39 kDa

37 kDa

GAPDH

GAPDH

39 kDa

IκBα

37 kDa

GAPDH

**Figure 8A**. The bottom two rows of bands were used for the figure in the manuscript. The higher row is IκBα; the lower row is GAPDH.

**Figure 8B**. The top row of bands is IκBα; the bottom row of bands is GAPDH.


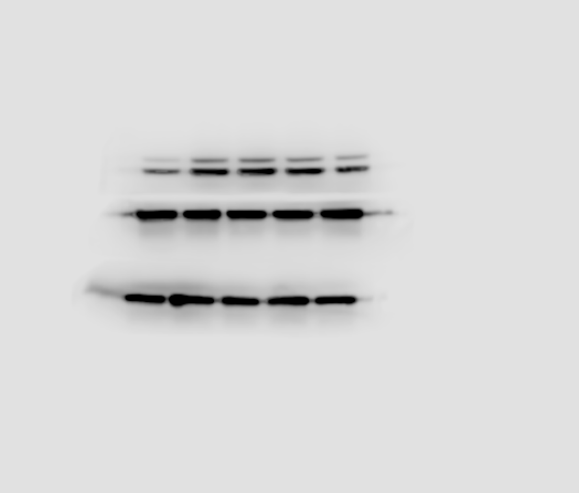

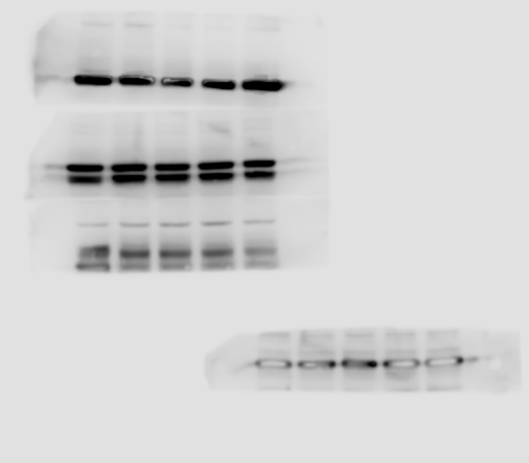

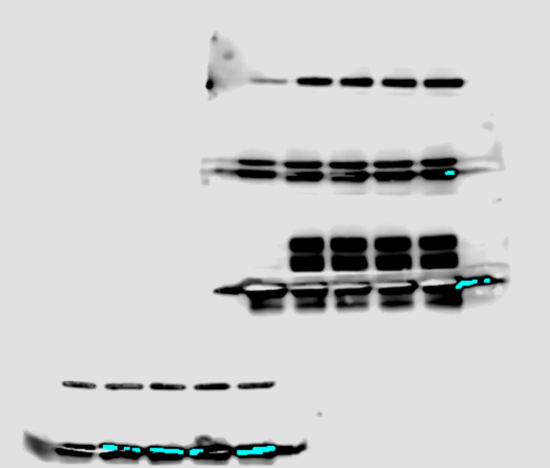


**Figure 10**. As indicated, the top row of bands on the left is p-p38 MAPK and the indicated doublet row is p-JNK. The top row of bands on the right is p38 MAPK; the middle row of bands on the right (doublet) is ERK1/2; the bottom row of bands on the right (doublet) is JNK. The first row of bands on the bottom (doublet) is p-ERK1/2 and the second row of bands on the bottom is GAPDH.

46, 54 kDa

JNK

42, 44 kDa

ERK1/2

37kDa

42,44 kDa

43 kDa

46, 54 kDa

43 kDa

p-ERK1/2

GAPDH

p38 MAPK

p-JNK

p-p38 MAPK
